# Dismissals for Social Media Hate Speech in South Africa: Animalistic Dehumanisation and the Circulation of Racist Words and Images

**DOI:** 10.1007/s11196-022-09937-y

**Published:** 2022-10-30

**Authors:** René Cornish, Kieran Tranter

**Affiliations:** School of Law, Queensland University of Technology, Brisbane, Australia

**Keywords:** Racial hate speech, Social media misconduct dismissals, Animalistic dehumanisation, Free speech, South Africa

## Abstract

Social media is changing the way humans create and exchange information. Not all social media communications are, however, civil: the ‘dark side’ of social media cultivates various ‘anti-social’ exchanges including hate speech. Parallel accelerating social media use has been an increase in decision-makers having to consider the legalities of dismissing employees for social media misconduct. This paper through an analysis of first instance South African employee dismissal decisions, identifies an economy of hate within South African workplaces. In 30% of social media misconduct decisions (120/400), employees were dismissed for circulating racialised hate speech. This hate speech took three forms. First was the use of animality discourse and animal metaphors to dehumanise colleagues and employers. Second, employees used words that had specific racist connotations within South Africa. Third, there was the direct deployment of signs or symbols connected with South Africa’s racialised past.

## Introduction

In South Africa, the 1994 elections promised a democratic nation infused with the hopes of unity, diversity and inclusivity—‘Simunye—We are one!’^1^ A metaphorical ‘Rainbow Nation’ representing a heterogenous society.^2^ However, racism and the vestiges of a colonial and apartheid past persist in South African society, economy and its public and private discourses [12: 671, 48: 22, 92: 377–378, 86: 325]. Racialised hate speech has been identified in South African online digital spaces [123: 53–68; 167: 42]. It has been suggested that racialised speech is the most prevalent form of hate speech on social media platforms [130: 89]. However, it is not just the verbal or textual expression of ‘words’ that can denote hate [75: 241]. Graphic images in the form of pictures and metaphors, or objects such as flags can also be considered hate speech [142: 446–447, 45: 309–332]. This paper explores how animal metaphors, graphic images and the symbols associated with the apartheid regime are decoded and deciphered as referents of ‘hate’ within the contemporary South African employment law context.

Located in the law and society tradition, this paper examines the legal texts (the dismissal decisions) as ‘social records’ or ‘archives’ evidencing expressions of hate rather than doctrinal records of the black letter law [32: 17, 195: 204, 198] on ‘hate speech’ as prescribed in the statutes. This paper argues, through a content analysis of first instance South African employment decisions, that employees utilise social media to circulate racialised hate using words and signs with cultural signification or historical connotations. This paper is advanced in three sections. The first section frames and contextualises the analysis by locating it at the intersection of hate speech, the semiotics of hate, the South African legislative framework and the employment law context. The second section presents the method, explaining how the sample of decision was identified, coded and analysed. The third section provides the findings. There were three. First the use of animality discourse and animal metaphors to dehumanise colleagues and employers. Second, the use of words that had specific racist- connotations and legacies within South Africa, and third direct deployment of signs or symbols connected with the apartheid regime.

## The Semiotics of Hate Speech, the South African Legislative and Contextual Framework and Social Media Misconduct Dismissals

‘Hate Speech,’ whether online or in the physical, is not a ‘universally defined concept’ [57: 4, 120: 325, 196: 56]. Cassim characterises ‘hate speech’ as ‘the use of abusive, racist and disparaging comments, words or phrases directed against particular race, religion, ethnic background, gender or sexual preference’ [25: 309]. In South Africa, ‘everyone has the right to freedom of expression,’ which includes ‘the freedom to receive or impart information or ideas’ as enshrined in the *Constitution* [31] by the Bill of Rights.^3^ However, the ‘advocacy of hatred that is based on race, ethnicity, gender or religion, and that constitutes incitement to cause harm’ is expressly excluded from the general right to freedom of expression under s 16 of the *Constitution of the Republic of South Africa* [31: s 16 (2)(c)].^4^ Hate speech and racial slurs are furthermore prohibited in terms of the *Promotion of Equality and the Prevention of Unfair Discrimination Act* 2000 [163: s 10 (1)(c)]^5^ and the *Employment Equity Act* 1998 [47],^6^ while the *Prevention and Combating of Hate Crimes and Hate Speech Bill* [160] is currently pending.^7^ Moreover, South Africa has various obligations in terms of international instruments.^8^ Under the South African legislative framework, the use of hate speech may therefore be understood as the use of ‘words as weapons’ which ‘inflict injury’ [200] rather than imparting ‘information’ or ‘ideas’ which is constitutionally guaranteed [31: s 16(1)(b)]. Jeewa and Bhima summarise the legal position that racialised speech may be considered speech of ‘no-value’ or ‘negative-value’ [86: 329].

The legislative mechanisms for regulating employment relations and dispute resolution in South Africa are the *Labour Relations Act 1995* [98] and various *Codes of Good Practice* [64]. Under the *Labour Relations Act* and the *Code of Good Practice: Dismissal* [29], ‘employees’ may only be dismissed for a ‘fair reason’ (one of which includes misconduct) and through ‘fair procedure’ [98: ss 188 (1)(a), 188(1)(a)(i), 188(1)(b)]. *The Code of Good Practice: Dismissal* provides guidelines on where a dismissal for misconduct is ‘unfair’ [29].^9^ The *Labour Relations Act* provides processes for disputes arising from unfair dismissal allegations [98: ss 191–192]. Employees can challenge dismissals for hate speech in the Commission for Conciliation, Mediation and Arbitration (CCMA), or in the relevant bargaining councils^10^ or private agencies accredited by the Commission [98: s 127].^11^ Importantly, while the decisions of decision-makers are not legally binding in terms of the doctrine of *stare decisis*, arbitration awards are final and legally binding on the parties to the dispute [98: s 143].^12^

There is a ‘substantial diffusion of digital communication into the Global South’ which is ‘shaping and disrupting these societies, economies, and cultures’ [32: 18]. South Africa is especially experiencing the ubiquitous proliferation of digital communication technologies and social media usage. As at January 2022, 41.19 million South Africans accessed the Internet,^13^ with a very high average daily use time of 10 h 46 min [90: 22].^14^ Significantly, 28.00 million South Africans were active social media users [90: 16], having an average daily use time of 3 h 43 min [90: 22].^15^ These users noted that work-related activities were a reason for using social media platforms in 36.3% [90: 53]. Moreover, the current proliferation in mobile connectivity^16^ as well as the upsurge in social media usage during the Covid-19 health pandemic suggests that digital communications—and with that further dissemination of hate through social media—is likely to intensify in South Africa.

Heryono and Helmy note that the verbal or textual expression of ‘words’ is not the only way to signify hate [75: 241]. Graphic images, metaphors, or symbolic objects such as flags or national monuments can be nonverbal signs of hate [142: 446–447, 45: 309–332, 11: 193–196, 118: 33–41]. Legal processes and decision-makers are forums where cultural meanings and practices are recorded, perpetuated, and challenged. In South Africa, with its colonial and apartheid past, racially hateful speech is pervasive. As such, it would therefore be expected that South African employment dismissal decisions would record words, graphic images and objects of hate, and more particularly expressions of racialised hate.

## Racialised Discourse: Hate Speech in CCMA Decisions January 2010–July 2021, South Africa

This paper examined 120 decisions of the Commission for Conciliation, Mediation and Arbitration (CCMA) and various Bargaining Councils for social media misconduct dismissals concerning racialised hate speech between January 2010 and July 2021. These decisions are archived on publicly available and a fee-for-service repositories, namely the Southern African Legal Information Institute (SAFLII) [183] and Sabinet [178].

Various search terms were used to identify the sample. These terms included ‘social network,’ ‘social network platform’ or ‘social media’ together with the words ‘work,’ ‘employment,’ ‘labour law,’ ‘employment law,’ and ‘discipline’ to limit cases to social media misconduct in the employment law context. The initial search identified 743 decisions where employees were ‘disciplined’ for social media misconduct. ‘Discipline’ includes progressive disciplinary action short of dismissal, so the sample was refined by limiting the decisions only to ‘dismissals’ for social media misconduct. This resulted in 684 decisions. To further narrow the sample, names of specific social media platforms such as ‘Facebook,’ ‘LinkedIn,’ ‘Twitter and social messenger applications with ‘group chat’ functions such as ‘WhatsApp’ and ‘Facebook Messenger’ were used. This sample was again revised for false positives, resulting in a sample of 435 decisions. Finally, all erroneously duplicated decisions were disregarded, resulting in 400 contested social media misconduct dismissal decisions in South Africa between June 2010 and June 2021. The ‘dismissal decisions’ sample was then analysed through a content analysis that included several readings of the decisions to identify major themes, followed by the subsequent extraction of relevant data from the decisions coded according to these themes. Several themes presented. Significant themes included employees using social media as channels to express online dissatisfaction and dissenting voice, to circulate gendered and sexual harassment, and for the perfusion of cyberviolence. One of the leading themes which emerged from the sample was that South African employees used social media to circulate racialised hate speech and signs with hateful connotations in 30% (120/400) of all the contested social media misconduct dismissals identified. It is these 120 dismissal decisions which formed the primary data source and ‘sample’ for this paper.

There were three significant findings that emerged from the sample. First the use of animality discourse and animal metaphors to dehumanise colleagues and employers. Second, the use of words that had specific racist connotations and legacies within South Africa, and third direct deployment of signs or symbols connected with the apartheid regime. Each of these are considered in detail in the next section. In addition, there were several general findings from the sample. Of these general findings, three are pertinent to contextualise and explain the main findings. The first was the portrayal of a racial group using ‘othering’ pronouns to denigrate, dehumanise and to indicate a ‘separateness.’ The second was the continued use of racial classifiers in post-apartheid South African workplaces, and the third was attempts by employees to make freedom of speech defences.

‘Othering’ is ‘the simultaneous construction…[i]n mutual and unequal opposition of the superior self or “in-group” in contrast to an inferior other or “out-group”[14: 70, 235: 1]. This opposition ‘reinforces notions of …[n]ormality’ of the self, and the ‘differences of others as a point of deviance…[which] effectively creates a separation between “us” and “them”’ [65: 1933, 132: 23, 196: 60]. ‘Online othering,’ according to Harmer and Lumsden, refers to a variety of ‘power contestations’ and ‘abusive behaviours’ which are ‘manifested on/through online spaces’ [67: 2]. Online ‘othering’ language in the form of pronouns to collectively refer to a group or demonstrate a ‘separateness’ from the employee was evident in 6% (7/120) of cases in the sample. One such decision was that of *Jikela v Smit Amandla Marine* wherein the employee’s Facebook post read, ‘…[t]here is nothing that disgust me like white people who always assume *they* know better, their “way” is better… You know actually that attitude that feeds their ill-founded superiority the one that makes *them* think *they* are “in charge” of black people …’ (emphasis added) [87: Phillips J [8]]. Similarly, referring to management as ‘*those* white people’ in *SACCAWU obo Mmoso v Mount Amanzi Holiday Resort* [179: Sikwane L [7], [17]] and to colleagues as ‘*these* White people…[t]ell *them* anything’ in *EAMWUSA obo van Duncan Wyk v Dart Stationers* further depicts the use of online ‘otherling language’ [44: Madotyeni Z [17]] (emphasis added).^17^

A second general finding, as suggested in the use of the collective ‘white’ in the othering decisions, was deployment of the racial classification system that was the bureaucratic cornerstone of the apartheid regime [13: 195], and endures in post-apartheid South Africa through the use of historical racial labels [116: 255]. Notwithstanding the repeal of blatant racist legislation^18^ and the passage of legislation that expressly prohibits unfair discrimination and advances equality,^19^ employees nevertheless used racial classification labels based on skin colour such as ‘white,’ ‘black,’ ‘Indian’ or ‘coloured’ to refer to individuals in 48% (58/120) of the sample.^20^ In *Roose v Netcare 911* [172], the employee posted a comment on Facebook which read, ‘Dear Blacks, If you find whites coming to your shacks to rob, rape or murder you…Or you find whites burning down your schools, busses or trains…Or stoning your cars and defacing your statues…You are most welcome to be racist, Sincerely Whites’ [172: Koorts M [18]]. In this instance, the decision-maker noted that ‘the racist comments were directed at all back persons’ as a collective group, were of an ‘inciting nature’ and had a ‘wider impact on employees’ and workplace harmony [172: Koorts M [70]-[71]]. The employee was not entitled to any relief sought, as the dismissal was found to be substantively fair [172: Koorts M [75]–[76]].

Employees raising the rights-based defence of freedom of speech was another feature in 12.5% (15/120) of the sample.^21^ Except for a single outlier, *Alexander v Ebesa Architects* [2: Madotyeni Z], all the freedom of expression defences were unsuccessful. The defence was unsuccessfully because the freedom of expression does not enjoy ‘superior status’ above other entrenched rights in the South African Constitution, but instead, there is a balancing of the fundamental rights of dignity, equality and freedom of speech [86: 330, 103: Yacoob J [44]–[47], 93: O’Regan J [25], 175: Kriegler J [41]].^22^ Mchangama and Alkiviadou note ‘it appears that South African lower courts tend to attach higher weight to dignity and equality than freedom of expression, when these values are seen to clash’ [125: 577].

In *Chiloane v Trans Africa Projects,* the decision-maker noted that ‘the right to freedom of expression does not extend to propagating hate speech, racist remarks and the impairment of the dignity of others’ [27: Kona T [20]]. ‘Hate speech,’ according to the decision-maker in *NUFBWSAW obo Liebenberg v Institute for the Blind,* ‘is not protected by the Constitutional right to freedom of expression and is entirely unacceptable in the workplace in any form’ [145: Jooma L [15]].^23^ This sentiment was echoed strongly in *Mahlangu v Chabo Joubert Air Conditioning Services* [111: Lekgwathi E]*.* The employee’s defence was that the use of the words ‘help the white genocide,’ ‘the only white man you can trust is a dead white man’ in his social media post was protected by the freedom of speech [111: Lekgwathi E [17]]*.* The decision-maker found that the employee had ‘read the Constitution of the Republic incorrectly’ [111: Lekgwathi E [17]]. The words were ‘clear, unequivocal and overtly racist in nature’ [111: Lekgwathi E [16]–[17]]. However, in *Alexander v Ebesa Architects* [2: Madotyeni Z], the employee’s reference to crooked whiteys [2: Madotyeni Z [14.1.2]] was held to be an expression of the employee’s views, and ‘acceptable free speech’ [2: Madotyeni Z [65]]. The decision-maker posited that the freedom of speech is ‘a constitutional protected right that should not be lightly interfered with’ [2: Madotyeni Z [71]] nor should it ‘attract any penalties from his employer’ [2: Madotyeni Z [65]].

These general findings reveal three core characteristics of the sample. First, the decisions manifest hate and racial speech by employees. Second, that within South Africa, race and hate, through ‘othering’ pronouns perpetuating apartheid-era racial categories endures. Third, that the first instance decision-makers generally did not excuse employee racial hate on freedom of speech grounds. Having established these general features, particular focus can be given to the findings of animality discourse, South African specific racist words and direct deployment of signs or symbols connected with the apartheid regime.

## Findings: Animals, Racist Words and the Symbolic Legacy of Apartheid

### Animalistic Dehuminisation and Animal Metaphors

The first significant finding was the utilisation of disparaging animal metaphors and animal imagery in 23% (28/120) of the decisions. In South Africa, Swart argues that ‘metaphors matter’ [211: 91]. Metaphors as ‘visual images’ [142: 446–447] are ‘general semiotic mechanisms’ or ‘vehicles’ which depict information about society and culture [216: 105–107, 229: 127]. Animals are often used as metaphors: ‘[a]nimals are effective vehicles for embodying highly emotionally charged ideas’ [213: 457, also 99: 301]. Haslam, Holland and Stratemeyer observe that animal metaphors offer a ‘rich metaphorical domain’ which may be used to either ‘praise’ or ‘vilify;’ ‘humanise’ or ‘dehumanise’ [69: 103]. ‘Dehumanisation’ refers to the denying of complete humanness in the ‘other’ [107: 759, 223: 65]. ‘Metaphor-based’ or ‘animalistic dehumanisation’ encompasses the association of the human to animal [107: 752, 71: 132, 72: 262, 76: 269] and often involves the perception of the ‘other’ as ‘primitives,’ ‘savages’ or ‘brutes’ [71: 132, 186: 91–92]. At the extreme spectrum of animalistic dehumanisation, the ‘superior’ group target the ‘other’ as being more animalistic than fully human, undeserving of human dignity: ‘vermin’ that need to be ‘exterminated’ [173: 86, 230: 109, 218: 216, 97: 487]. Violence and genocide against the ‘sub-human’ ‘racialised others’ is justified [185: 152, 33: 159]. The animalistic dehumanising of Tutsis who were reduced to ‘cockroaches’ by the Hutu in Rwanda and the Nazi propaganda portraying the Jewish peoples as ‘rats’ or ‘vermin’ manifest this tendency [230: 109, 173: 86, 69: 94–95, 60: 293]. The brutality justified against the ‘animal’ is imposed on the ‘animalised:’ they ‘simply destroy’ and so ‘must be destroyed’ [33: 169, 218: 216]. A particular iteration of animalistic dehumanising is ‘simianisation’ where the denigrated human is considered like an ape or monkey [232: 104, 201: 79].

Simianisation has a protracted and ubiquitous association with black bodies particularly from Africa [211: 91, 232: 105, 201: 78, 82: 8–10, 85: 44–49]. Indeed, animal metaphors have been identified as deep-rooted in the South African colonial and apartheid ‘bio-necropolitical systems’ [35: 74]. In colonial discourse, African bodies were depicted as the nonhuman animal ‘other’ [212: 62, 42: 130]: ‘brutish savages’ or ‘wild beasts’ to be ‘conquered’ [217: 42, 133: 204]. Kim’s ‘race-species meanings’ frames the ‘human’ as ‘white’ [35: 74]. Human and animals are resultantly in ‘oppositional imaginary;’ ‘blackness’ and ‘humanness,’ were according to Mbembe, ‘ontological impossibilities’ [122: 54, 35: 77–78]. To be human, is to be ‘not animal’ [155: 446]. To be ‘white’ is to be ‘not wild’ [155: 446]. However, animal metaphors are not only employed by the ‘oppressors’ ‘down the power gradient’ to subjugate and dehumanise dispossessed peoples [18: 2]. Bruneau and Kteily suggest that dehumanisation also occurs ‘bi-directionally up and down the power gradient’ [18: 2]. In South Africa, previously oppressed, historically disadvantage persons also used metaphors against their former oppressive ‘baas,’ suggesting that racism and racialised hate speech circulates between various cultural actors. In some instances, a dualist attack is waged: the integration of both ‘dehumanising’ and ‘disgusting’ elements through which the attacker adds an extra layer of repugnance and repulsion to the hateful communication [76: 277].

The sample records broad use of animalistic dehumanisation and animal metaphors. An employee accused an employer of believing ‘blacks are animals to make profit’ [136: Sithole N [19]]. Employees also made direct, stereotypical racist posts. In *Harvey v Little Gems,* the employee referred to children in their care as ‘dog eaters’ [68: Molefe E [10.30], a derogatory reference to individuals of ‘Asian descent’ [222]. In some decisions, the employee obliquely engaged with animal imagery through references to proverbs or idioms. In *Mosala v Fidelity Security Services,* the employee used the metaphorical figure of speech ‘tail between his legs’ when alleging that the employer was not *au fait* with the employment law [131: Basholo V [4.1.4]]. This expression alludes to ‘a dog that slinks off in defeat’ [52(a)].

However, the bulk of the sample was not as oblique. In many decisions, employees made direct connection between a racialised body and an animal. There were widespread examples of simianisation where black bodies were represented as ‘monkeys,’ ‘baboons’ or ‘apes,’^24^ and common portrayal of white or brown bodies were as ‘snakes,’ ‘dogs’ and ‘pigs.’^25^ Simian and canine metaphorical expressions are grounded in ‘degradation’ and dehumanisation [69: 94, 70: 318], while the use of ‘offensive’ animal metaphors of ‘disgusting’, ‘reviled’ or ‘taboo’ animals such as ‘pigs,’ ‘snakes’ or ‘rats’ seemingly involve a symbolic transference of the ‘reviled’ characteristics of ‘depravity and disagreeableness’ from animal to human rather than the denial of humanity [69: 94, 70: 318, 71: 135].

#### Black Bodies and Simianisation

Animalistic dehumanisation, ‘simianisation’ and the animalising of black bodies through metaphorically likening these individuals to ‘baboons,’ ‘monkeys’ or ‘apes’ reveals racialised animality discourse in South Africa [3: Khalil, 58: 2, 117: 25–64, 233: 65]. Yet, animalistic ‘othering’ is not unique to South Africa [211: 91]. While primate metaphors have universally been used as ‘mechanisms of social control,’ [211: 99] the potency of the ‘monkey metaphor, the ape analogy and the simian simile,’ according to Swart, lies in the ‘local forms and inflections’ of ‘othering’ in the South African milieu [211: 90].

In African settler history, ‘baboons’ have always been associated with ‘vermin’ [210: 54]. However, before settler colonisation of southern Africa, traditional rock art demonstrates that indigenous groups did not depict the baboon as animals of ‘contempt’ [211: 94]. Instead, for some indigenous peoples such as the Ncube, baboons were ‘constitutive of their personal identities:’ ancestorial, ‘totemic’ animals [211: 94, 88: 85–103].Others, such as the San tribes, did not experience baboons as the ‘enemy,’ but bestowed on these primates a ‘quasi-human position, replete with agency’ in San ‘cosmology’ [211: 94, 88: 85–103]. Baboons were revered [211: 94]. Nor were baboons always encountered as the enemy [211: 95]. The hostile association between humans and baboon emerged not only through racist colonial discourse, but was augmented by the threat these primates posed to the crops and livestock of agrarian, coloniser farmers [211: 94, 233: 65]. Today, legislated as ‘vermin’ in South Africa, baboons live on the fringe of the ‘wild’ and ‘civilised’ human society [233: 66].Woodward notes that this is seemingly ‘symbolic’ of their place in the ‘evolutionary’ scale—at the intersection between humans and other nonhuman primates [233: 65]. The extreme hatred and brutality levied towards baboons in South Africa suggests that the disproportionate violence is more about ‘societal anxiety in general;’ the primate presenting as ‘human proxy’ [157: 131, 211: 97] having ‘traits of human society’ cast onto their non-human, animal bodies [59: 21]. In this instance, the simian metaphor is seemingly ‘reversed’ [59: 20–21, 9: 38–47]. De Robillard and Lipschitz note that post-apartheid, there have been several occasions of farmers discharging loaded weapons at black bodies, maintaining that they thought they were ‘shooting at baboons’ or ‘monkeys’ [35: 76–77]. In these situations, black bodies have been ‘bloodily and bodily linked’ [104: 16] to the ‘killable animal’ [35: 77, 79].

Simianisation has a protracted and ubiquitous association with black bodies [232: 105, 82: 8, 201: 78, 85: 44–49, 211: 91]. The primate metaphor associated with dark-skinned bodies is deep-rooted in the colonial era of ‘racial anthropology’ [119: 24] and the ‘race science’ [211: 95] that argued that Sub-Saharan Africans were not fully evolved [69: 96, 60: 293, 201: 93–94] but ‘simianised’ [63: 141, 71: 135]. Described as being ‘a hair’s breadth away from nonhuman primates’ [201: 78] and as ‘liminal figures at the edge of humanness’ [211: 94], persons of African descent were ranked somewhere on the ‘Great Chain of Being’ [201: 85] or ‘evolutionary scale’ [119: 25] between the least developed ‘simian’ and the marginally more advanced ‘savage and/or deformed anthropoids’ [60: 293]. While dark bodies were categorised as ‘incomplete’ [188: 292] or ‘less completely’ human [186: 104], white bodies were ranked the highest and most evolved of beings [60: 293]. Martin notes that ‘the ape became a vehicle through which to express colonial racism’ [119: 24]. The colonial settlers segregated and subjugated the ‘almost but not quite human’ colonised peoples of Southern Africa and ‘legitimised’ the racialist practices through association of the indigenous dark-skinned bodies with apes [119: 24, 211: 92]. The animalised simian metaphor became utilised as a form of controlling the ‘uncivilised, primitivist’ ‘African behaviour’ [211: 96].

The ‘baboon’ metaphor, which was employed both during colonial and apartheid discourses as a racist insult [233: 65], has a complex history [211: 95]. While the English did liken the Boers to ‘baboons’ [214] it was the use of the dehumanising, animalistic metaphor for peoples of African descent [211: 95] which ‘collocated’ the baboon with indigenous groups living in Southern Africa [233: 65]. In this way, ‘monkeys’ and ‘baboons’ became the ‘lightning rod’ for racial segregation between black and white bodies and were then ‘fashioned into instruments of insults’ [63: 138, 141]. What emerges from the sample is that this metaphorical construct continues to circulate in post-apartheid South Africa. In contemporary South Africa, the ‘monkey,’ ‘ape’ and ‘baboon’ metaphors have the power to ‘shock’ and ‘wound’ [211: 96].

Traditionally, ‘ape’ has been associated with ‘a stupid,’ [59: 26] ‘uncouth’ individual [129(a)] or ‘a large, awkward, unattractive, and uncultured male’ [151: 7]. In the South African context, the ‘monkey’ conveys the ‘explicit message that black people are not worthy of being described as human beings’ and that dark-skinned bodies had ‘subhuman’ or ‘low intelligence’ [3, 21: 210]. The reference to the South African government as ‘fucking stupid monkeys running our country’ in *Cantamessa v Edcon Group* [23: Khumalo B [40], [46]] and the distribution of an image of a ‘monkey’ with Afrikaans words translated as: ‘I wonder what the ‘volk’^26^ (interpretated in this context as ‘black people’) are burning today?’ in *PSA obo O'Kelly v SARS* [164: Terblanche M [4], [9]] are two examples of dehumanising simian imagery circulated by employees in the sample. Some of the recipients as well as the employer deemed the image as ‘having racial connotations’ as ‘history had shown that blacks were referred to as monkeys by white people’ [164: Terblanche M [4], [11]]. The decision-maker noted that ‘in terms of the South African history and apartheid era, there have been several examples of racism by the use of the word monkey in reference to black people. This is not an opinion, but a fact’ [164: Terblanche M [26]]. These decisions support De Robillard and Lipschitz’s argument that ‘animals and race circulate to restage a racist past and anxious present’ in the post-apartheid South African milieu [35: 81].

A significant feature of the sample was that employees on several occasions posited that the online ‘monkey’ comments were not ‘racist’ but used the ‘disclaimer’ that they were merely ‘jokes,’ ‘banter’ or were said in ‘jest.’ Hodson, Kteily and Hoffarth [76: 267–268] and Hodson, Rush, and MacInnis [77: 660–682] have noted that ‘jokes’ and ‘dehumanising metaphorical speech’ can be a means of conveying ‘negative intergroup sentiments.’ Race-based amusement and racist jokes which depict racial or ethnic ‘others’ as ‘inferior’ or ‘stupid’ are a ‘cultural tool’ that dehumanises dark-skinned bodies, divides social groups, perpetuates inequalities and symbolises the ‘dark side of humour’ circulating in society [156: 970, 958]. Dark-skinned bodies are therefore considered as ‘figures of fun,’ mockery and ridicule [106: 813].

The decision-maker in *Numsa obo Ncikazi v Express Employment Professionals* noted that ‘[r]acism can take many forms, it can also include hatred because one believes that the other race is inferior…[i]t is not always expressed in violent or intimidating behaviour, racial name calling and racist jokes also constitute racism [146: Ngwenya V [33]]. Significantly, in *Naidoo v Illovo Sugar*, the decision-maker stated that it was ‘irrelevant’ that some individuals found the post which had ‘extreme racist connotations … funny’ [134: Whitear-Nel N [5.1.2], [4.6.4]]. At best, the defence that such racist comments were said ‘in jest’ would serve merely as a ‘mitigating factor’ [134: Whitear-Nel N [5.1.3]].

Another example is *Hoskins v Standard Insurance Limited* where the employee posted an image to a social media group which included work colleagues [79: Deysel A [11]]. The picture depicted a large, seated primate with a small black child on either side, holding its hands on the children’s shoulders in a ‘fatherly manner.’ The caption read, ‘Sit down kids. I will now tell you how I met your mother’ [79: Deysel A [12]]. The employee believed the picture to be ‘humorous’ and sent it to his colleagues ‘with the intention to solicit some laughter from them’ [79: Deysel A [13], [31]]. The decision-maker noted that ‘[s]ending a picture depicting black children as the offspring of a primate to a racially diverse group of work colleagues and implying that there is something humorous about it, is racist, insulting and offensive’ [79: Deysel A [34]].

Likewise, in *Naidoo v Illovo Sugar,* the employee sent a picture of three monkeys with a caption ‘there are four monkeys looking at each other.’ The picture insinuated that the individual looking at the screen was the fourth monkey [134: Whitear-Nel N [3.2]]. A colleague believed the image ‘referred to the apartheid era where it was a common racial slur to refer to black Africans as monkeys,’ and testified that his ‘offense and hurt ran deep’ [134: Whitear-Nel N [4.1.5]]. The decision-maker found that in South Africa, ‘such an image would have the very real potential to cause offence, particularly to black people’ [134: Whitear-Nel N [4.6.3]]. She further stated that ‘[i]t is trite that referring to black Africans as monkeys has extreme racist connotations, harking back to the atrocity of white supremacy and the policy of apartheid’ [134: Whitear-Nel N [4.6.3]].

‘Bigots try to hide behind terms like “it was made as [a] joke” and that there was no malice involved’ [168: Mooi F [32]]. Lockyer and Pickering argue that racist humour is in fact a style of ‘comic malice’ [106: 811]. Using the ‘only joking’ humour ‘disclaimer’ or ‘classic let-out clause’ for racist animality discourse presumes that ‘a joke’ cannot be unfunny, nor can it be uttered with ‘serious intent’ [106: 812, 80: 46–47, 7: 42]. The sample shows that decision-makers were not convinced by the ‘just joking’ rhetoric. The sample further reveals that it is the ‘moral defectiveness’ of animalistic dehumanising racial ‘jokes’ which renders them no laughing matter [24: 546]. In this sense, dehumanising metaphorical speech masquerades as ‘humour,’ and simply put, is a vehicle for circulating racialised hate.

#### White Bodies and Brown Bodies: Canines, Rodents, Snakes and Swine

Animal metaphors used to denigrate white and brown bodies were generally ‘negative’ or ‘uncomplimentary’ [69: 101, 203: 243, 245, 247]. ‘Unclean,’ ‘disgusting’ or ‘taboo’ animals such as snakes, rats and pigs seemingly involve a symbolic transference of the ‘reviled’ characteristics from animal to human [232: 105, 69: 94, 71: 135, 70: 318]. The sample revealed that the animal discourse used for white and brown bodies included dogs, snakes, pigs, prawns and rats.

Dogs have ‘symbolic currency in human language’ [62: 149]. The dog, ‘man’s best friend’ [212: 62, 154], often symbolises desirable human qualities such as fidelity [152: 264] and loyalty [81: 137, 212: 62].^27^ However, ‘dog’ can also epitomise the ‘dark side of human nature’ [212: 61] and has very negative connotations within South Africa. Canines depict the ‘diabolical’ or ‘ominous’ in much of South African literary works, particularly when used in the figurative or metaphorical sense [212: 61]. While canine metaphors do have negative denotations [69: 94] (reference to a ‘worthless,’ ‘despicable,’ [81: 11] ‘unpleasant’ or ‘untrustworthy’ person [59: 26, 154], in South Africa, the use of dog invectives, particularly ‘*inja*,’ surpasses mere insult [5: 345]. In the sample, references to disparaging canine metaphors or imagery for white and brown bodies was evident in 3% (4/120) of the decisions.^28^

The isiZulu word ‘inja,’ according to the Dictionary of South African English, is a ‘derogatory term of contempt’ [40 (f)]. In *Ntshangase v MCFI International SA,* the employee posted a comment on Facebook in isiZulu that translated as, ‘My white boss who I am working for is a dog (‘*inja*’), together with his loyalists and spies’ [143: Jazbay SA[18]]. The employer testified that the word ‘inja,’ ‘meaning dog,’ ‘is deeply disrespectful and offensive in Zulu culture,’ and combined with other posts ‘wish[ing] someone would sleep with the boss’s wife’ and that the ‘boss must be sodomised’ constituted ‘hate speech’ [143: Jazbay SA [19]–[20]].

**Fig. 1:**
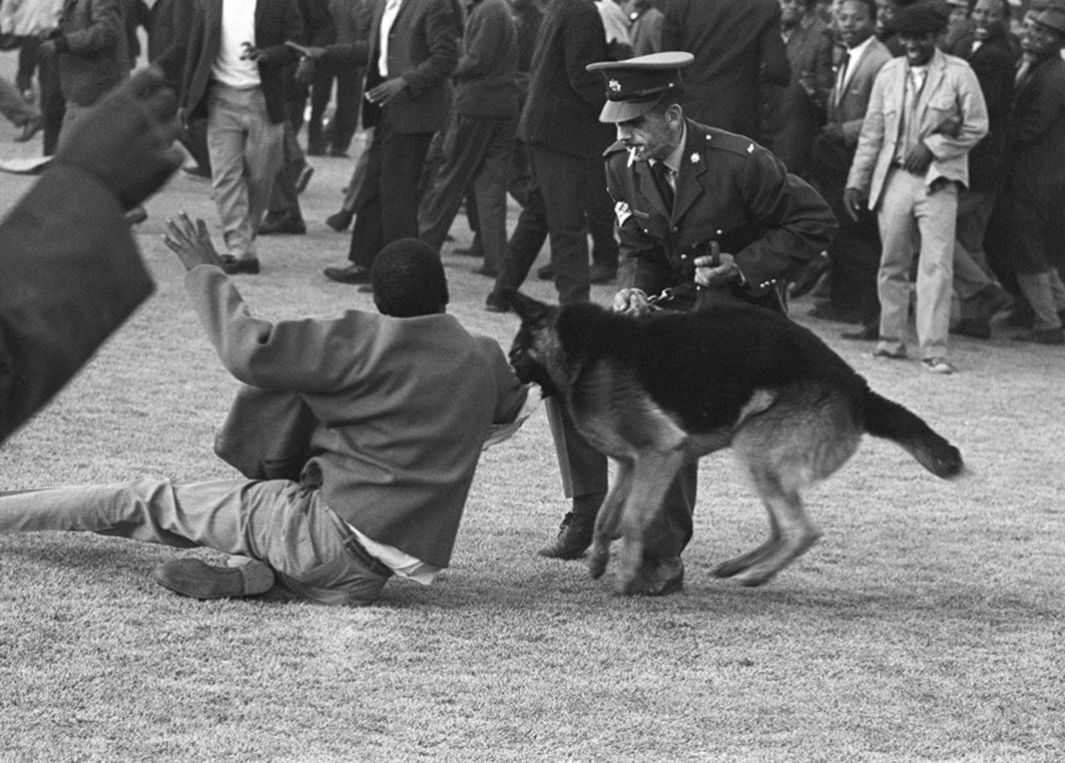
Alf Kumalo/Africa Media Online. Police and dogs at Orlando Stadium, 1960s

**Fig. 2:**
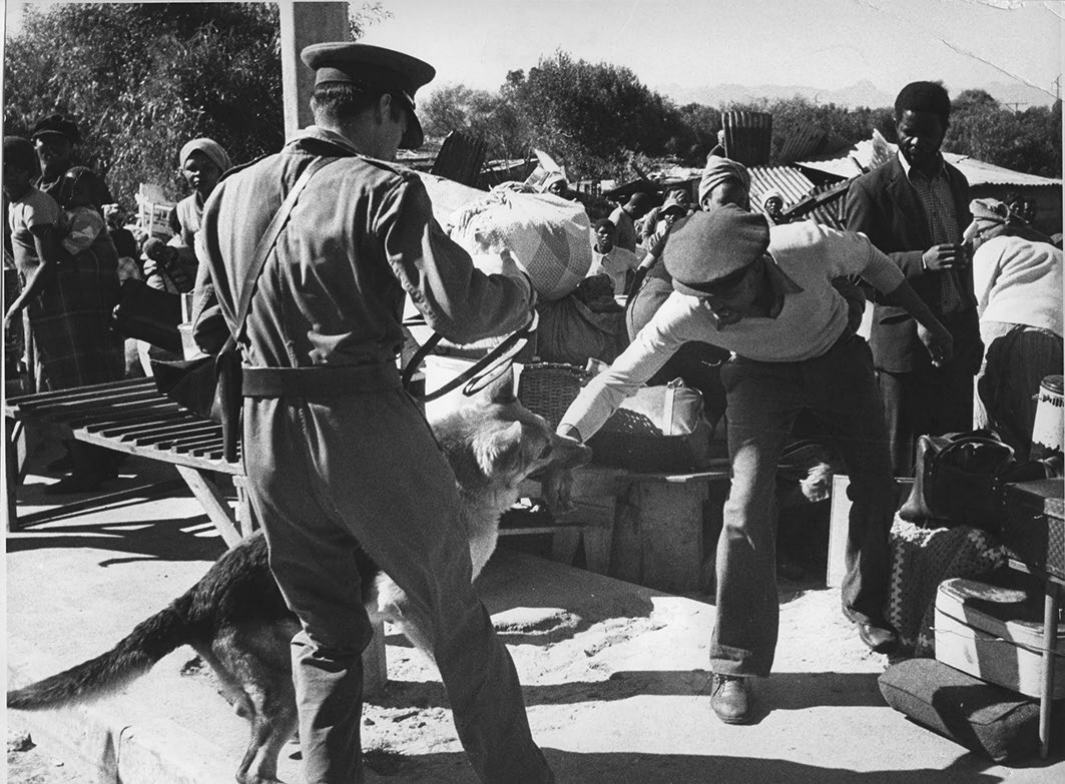
Peter Stanford/African News Agency/ANA. Police and dogs out in force during bulldozing of Modderdam Road squatter camp

In the South African context, Baderoon notes that ‘canine insults’ have been used for many years in South Africa to ‘naturalize ideological differences and justify aggression’ [5: 345]. Similarly, Ndebele argues that dog imagery has been ‘a pervasive symbol of …[v]iolence’ and ‘abuse,’ conjuring images of ‘denigration and brutal punishment’ under colonialism and apartheid rule [137: 2, 4]. More particularly, the police dog symbolised an instrument of the apartheid regime—the ‘weaponised’ embodiment and metaphorical expression of apartheid [224: 30, 225: 160]. The use of dogs was ‘emblematic of Africans’ experiences of the white-supremacist state’ [191: 195, 192: 143–163]. This can be forcibly seen in Figs. 1 and 2.

Employees also made disparaging references to brown bodies as ‘rubbish dogs’ in *Shozi v Standard Bank* [194: Lyster R [5]] and as ‘p*** hont’ in *Watson v SBV Services* [226: Dhlodhlo P [30]]. The word ‘hond’ is the Afrikaans noun meaning ‘dog’ [231 (a)]. ‘P****’ is the derogatory term for female genitalia [81: 113]. Hodson, Kteily and Hoffarth explore the integration between ‘dehumanisation’ and ‘disgust’ which they propose are ‘natural bedfellows’ [76: 277]. When individuals exploit the ‘double-pronged attack’ which is derived from the simultaneous dehumanising of the victim and the evoking of disgust, the hateful communication, the authors argue, is even more abhorrent [76: 277]. The reference to ‘dog’ is to ‘dehumanise:’ to perceive these bodies as ‘subhuman’ [76: 277]. Through the inclusion of the accompanying epithet ‘rubbish,’ meaning ‘garbage,’ ‘worthless,’ or ‘unwanted’ thing [22], the attacker adds that extra layer of repugnance and repulsion to the hateful communication [76: 277].

Similarly, referring to a female employer as ‘bitch’^29^ in *Kalik v Woolworths* [89: Mlaba N [19]] is also an animal metaphor [69: 92, 171: 77–100]. It is used as a derogatory ‘gendered insult’ [56: 16, 18, 25] or label for an ‘unpleasant’ or ‘difficult’ individual [30 (a)]. Kleinman, Ezzell and Frost argue that the word ‘bitch,’ which has ‘a non-human’ ‘female referent,’ is used to dehumanise women [94: 47, 58]. Weston-Sheuber notes that derogatory ‘gendered epithets’ which are only associated with women’s bodies, or which associate female bodies with ‘animals,’ such as the use of the word ‘bitch’ are so commonplace that these hateful words are often glossed-over or ignored as common parlance [228: 140].

As with ‘*inja*’ and primate terms, some animal terms, such as ‘*igundwana*’ (meaning rat) which do bear universal significations [69: 94], have especially negative connotations in the South African context [55: Rustin Q [36]–[37]]. Universally, the ‘human is rat’ metaphor is one of ‘deep contempt’ [81: 11] denoting ‘a disloyal, deceitful person,’ [59: 27] a ‘despised person’ or an ‘informer’ [203: 244, 151: 315]. The idiom, ‘to “rat” on someone’ refers to ‘informing on someone’ [151: 315, 52 (b)]. In South Africa, ‘rats’ have been associated with ‘*ubuthakathi,*’ [236] ‘*ukuthakatha*’ [231 (b)] or ‘witchcraft’ [19: 317, 41: 105] as the carrier of ‘curses’ [41: 105]. Many believe ‘rats’ are sent by ‘jealous’ or ‘malicious people,’ or individuals with ‘evil intentions’ to ‘spy’ on and listen to private conversations [19: 321, 41: 110–111, 4: 285]. This information is then exploited for harmful or nefarious purposes [19: 321, 41: 110–111, 4: 285]. In the sample, the reference to ‘*igundwana* (rat/rotte)’ was made in the context of a photograph of two non-striking managers working during protected industrial action being sent to union members with the words ‘the rot(te) are working’ [55: Rustin Q [37]].

In *Kalik*, the employee used a barrage of animal terms to denigrate management [89: Mlaba]. Calling superiors ‘pigs,’ ‘bitches,’ ‘prawns,’ and ‘donkey’ was deemed ‘derogatory, offensive, insulting’ and ‘rude,’ and was serious misconduct that warranted dismissal [89: Mlaba N [11], [19], [28], [63]]. The employee referred to a manager as ‘a prawn,’ included an image of a prawn and the description that a prawn had ‘no backbone, no guts and its head was full of shit’ [89: Mlaba N [29]]. Sommer and Sommer noted that non-mammalian zoomorphs were rare, [203: 237, 246] with fish metaphors being particularly uncommon [69: 92]. The ‘human as donkey’ metaphor means ‘silly, stupid person’ [59: 27] or ‘fool’ [203: 244]. The pig is considered in many cultures as ‘vile’ and ‘repulsive,’ the objects of ‘scorn’ and taboo’ [99: 315]. The ‘human is pig’ or ‘swine’ metaphor refers to a ‘greedy,’ ‘unpleasant,’ ‘difficult’ or ‘unkind person,’ [59: 27] but also to a ‘filthy,’ [151: 288] ‘dirty’ or ‘slovenly’ individual [203: 244]. The employee altered the profile picture of a manager by editing the image and ‘putting a pig’s nose on her’ and referred to her as ‘a pig’ [89: Mlaba N [29]]. Additionally, she then edited a picture of the manager’s spouse’s face (who also worked for the employer) and put a pig’s face on him [89: Mlaba N [23]]. Similarly, the use of a ‘swine-taboo’ by means of an incongruous simile was evident in *Leih v World Net Logistics* [101: Sosibo]. The employee had been issued a final written warning prior to dismissal for the comment, ‘…[t]hey are more confused than a Muslim stuck on lifeboat with only a pig for company’ [101: Sosibo L [105]]. The statement refers to the religious restrictions on the consumption of pork in the Islamic faith [105: 59].

The universal ‘snake’ metaphor denotes a ‘worthless, no-good’ [151: 354] ‘deceitful and unprincipled person’ [203: 244]. It is also a reference to an ‘unpleasant, untrustworthy person’ [59: 27]. ‘Viper’ similarly denotes ‘treachery’ [81: 11]. In South Africa, snakes are a ‘heavily-laden symbol’ with a powerful ‘cultural significance’ [4: 287]. Associated with witchcraft, the snake may be a ‘familiar’ sent by a witch to cause mischief and misfortune, or is given to an individual as ‘*muthi:*’ a creature is created by the witch, and when eaten either in a dream or with a meal, typically manifests in the human body as a snake which then consumes the person from within [4: 289]. In *Jikela v Smit Amandla Marine*, the employee posted that ‘…[n]othing grates my nipples like the underhanded tricks some insecure people use to make themselves feel like they are adding value in the corporate space. I despise snakes!…[T]he next thing when shit hits the fan they hide in their socks and slither off to their holes # Bloody snakes’ [87: [8]]. Further references to snake metaphors were evident in several cases including *Pieters v Southern Canned Produ*cts [158: [6], *Shayi v Quality Pourts* [190: [16] and *Shozi v Standard Bank* [194: [5]].

In *Mduduma v Jabulani Food Lovers Market,* referring to a colleague as ‘a foreign snake,’ which was deemed to be a derogatory and xenophobic term by the employer, the decision-maker found that the employee was ‘out of control, inflammatory, defamatory, a pernicious harasser and derogatory discriminator’ [127: Moni N [31], [48]]. In referencing xenophobic violence, De Robillard and Lipschitz note that ‘foreign bodies are made abject and resignified as animal,’ and this resignification in turn results in ‘a deadly slippage between metaphor and material bodies’[35: 74, 104: 16]. Through abjectification, foreign bodies are linked to all which is deemed ‘foul, impure and a threat …[w]hich must be violently expelled for the sake of the security, or indeed purity, of the national body’ [35: 75]. As a result, the ‘foreigner-as-animal’ is ‘bloodily and bodily linked’ to the abject formation of the killable: the foreign body is ‘inextricable’ from the unclean, the repulsive and repugnant, and the pestilent [104: 16].

In summary, this section advanced that ‘offensive’ animal metaphors of ‘unclean,’ ‘disgusting,’ ‘reviled’ or ‘taboo’ animals such as ‘pigs,’ ‘snakes’ or ‘rats’ were used to animalise white and brown bodies. The next part explores the cultural semiotics, semiotics of flags and visual imagery.

### South African Specific Racist Lexicon

The second distinct finding was the use of specific words in the South African lexicon which objectively seem to be racially neutral, bear ‘historical’ or ‘cultural’ connections that ‘qualify them as hate speech’ [114, 58: 26–31]. These racial slurs were part of the apartheid-era vocabulary used to denigrate and debase individuals, and their continued use is ‘offensive and demeaning,’ effectively ‘re-opening old wounds’ [168: Mooi F [169]]. Similarly, hate and intolerance find expression in ‘cultural artefacts’ rooted in ‘historical’ and ‘cultural contexts’ [185: 150]. The ‘historical context’ of ‘symbolic expressions’ of hate is ‘precisely what imbues them with meaning’ [229: 122]. In 21% (25/120) of the sample, employees used South African specific racist lexicon.

#### Ethnophaulisms for White Bodies

‘Ethnophaulisms,’^30^ derived from the Greek ‘ethnos’ (‘people’) and ‘phaulisma’ (disparage), are ‘pejorative’ names or words usually based on ‘observable phenomenon’ which are used ‘to deprecate a group of people' [144: 29]. These ‘ethnic slurs’ constitute hate speech [144: 29–30], particularly on account of their pejorative usage during colonial and apartheid eras [73: 2]. While most of the racial descriptors identified in the sample signify interracial hate, it is important to note that ‘white society’ in South Africa is also comprised of two ‘ethnocultural groups’, namely English-speaking and Afrikaans-speaking individuals (the latter commonly referred to as ‘Afrikaners’ [11: 183].^31^ Derogatory language is also used by white English and Afrikaans speakers to denigrate the ‘other.’ Common Afrikaans descriptors to allude to an Englishman, or English-speaking South African is ‘rooinek’ [40 (i)],^32^ ‘soutpiel’ or ‘soutie’ [40 (j)].^33^ Similarly, disparaging descriptors used by English speaking South Africans to refer to Afrikaans individuals include ‘Dutchman,’ [40 (d)], ‘rockspider’ or ‘rock’ [40 (h)] and ‘crunchie’ [40 (c)].

The term ‘baas’ in South Africa means ‘boss’ or ‘master’ [129 (b)].^34^ According to the Dictionary of South African English, ‘baas’ was used during the apartheid era to refer to ‘a white male,’ ‘indicating the speaker’s perception or acknowledgement of the other’s superior social status’ [40 (a)]. Today, it may be used ‘ironically,’ but is often deemed ‘offensive’ to be addressed as such [40 (a)]. Signifiers such as the term ‘baas,’ according to Enwezor, are established on ‘blackness as anathema to the discourse of whiteness’ [48: 22]. ‘Baas’ was evident in 2% (2/120) of the sample.^35^

Another derogatory racial descriptor for white bodies is the use of the term ‘boer.’ ‘Boer’ means ‘Afrikaner’ or ‘farmer’ [148: 104–105], and is considered ‘offensive’ and ‘demeaning’ [168: Mooi F [168]–[169]]. It has been noted that this term carries similar derogatory connotations to the ‘k-word’ [57: 27, 114]^36^ and its use has justified dismissal, even in instances where employees stated that such usage was acceptable in the workplace [226: Dhlodhlo P [47], 161: van der Merwe F [31]]. In the sample of the decisions manifesting racialised hate speech, 11% (13/120) of employees made specific reference to the term ‘boer.’^37^

The employee in *NUMSA obo Daniels v Polyoak Packaging* stated that ‘in the industry bosses and the police are referred to as ‘boer’[147: Erasmus T [61]]. Similarly, in *NASECGWU obo Nkomombini and Steyn Diamante,* the employee averred that the word ‘boer’ was used to refer to the ‘owner’ or ‘someone who was in charge,’ and that in using the word ‘boer,’ ‘he had not meant to offend anyone’ [135: Rabie M and Mosoma PS [20]]. The decision-maker, however, found that the employee had used ‘vitriolic and disgraceful utterances’ [135: Rabie M and Mosoma PS [41]]. Similarly, in *Booysen v Namaqua Wines SA,* the employee incited colleagues against management by stating that they had to ‘protect each other’ because for years the ‘boere’ had ‘been sowing division amongst them’ [10: Mohamed S: [4]]. The decision-maker noted that ‘racist comments and vulgar language cannot be tolerated in the workplace neither in society as a whole’ [10: Mohamed S: [20]]. Likewise, in *CEPPWAWU obo Van Wyk v Atlantic Oil,* the decision-maker found that where the employee referred to white people as ‘boertjies’ and ‘whities,’ was ‘racist in its context’ [26: Du Plessis JS [28]]. Encouraging people to stand up against ‘die boere’ was a statement which, according to the decision-maker in *Paulse v JD Kirsten Boerdery*, would ‘stir up emotions and debate’ and the damage which was caused when the comment was posted ‘was an egg that could not be unscrambled’ [121: Nash M [13], [36]].

In 3 decisions, reference was made by employees to ‘kill the boer.’^38^ In *FAWU obo Du Preez v Aanhalt Boerdery,* the reference was to ‘chase the boere into the sea’ and to ‘kill the boer, kill the farmer’ [53: Mgubasi A [12]]. Similarly, ‘kill the boer’ and ‘down with whites’ was used in *UCIMESHAWU obo Khumalo v Gooderson Drakensburg Gardens* [221: Ngcobo AB [4]]. In these decisions, the dismissals were upheld, as the posts used ‘inflammatory language’ [221: Ngcobo AB [5]] or were deemed ‘inappropriate’ [53: Mgubasi A [30]]. In *SDTU obo Liebenberg v Botha*, the decision-maker did not gloss over the employee’s reference to ‘‘n dooie boer is ‘n gooie boer’ (meaning ‘a dead boer is a good boer’), notwithstanding her justification that she did not mean the words ‘in an ugly way’ and understood it ‘to be a mere saying’ [189: Jooma L [9]]. The ‘derogatory statement,’ according to the decision-maker, amounted to ‘hate speech,’ and that ‘[g]iven the context of the history of South Africa, it is universally accepted that one does not make such comments as they are perceived and understood to impair the dignity of a section of the population’[189: Jooma L [15]]. Confirming that ‘hate speech’ did not enjoy constitutional protection under the ‘right to freedom of expression,’ the decision-maker further noted that hate speech was ‘entirely unacceptable in the workplace in any form’ [189: Jooma L [15]].

#### Ethnophaulisms for Black and Brown Bodies

Further in the sample there was evidence of ethnophaulisms to dehumanise and denigrate black or brown bodies. Brown bodies were referred to as ‘coolie’ or ‘koelie,’ which is an offensive, derogatory, insulting and hurtful term ‘for one of Indian descent’[153: 191, 123: 54, 38: 47–50, 168: Mooi F [167]–[169], [172], 40 (b)]]. Further slurs included ‘hottie,’ ‘hotnot’ or ‘Hottentot.’ ‘Hottentot’ is said to derive from the Dutch word ‘huttentut’ [81: 241] or German ‘hotteren-totteren’ meaning ‘stammerer’ or ‘stutterer’ [150], as the settler peoples found the ‘clicking sounds’ and ‘staccato pronunciation’ of the indigenous Khoikhoi language ‘strange’ and ‘bestial’ [123: 54, 108: Mamosebo J [20]]. ‘Hotnot’ and ‘hottie’ are the abbreviated forms thereof [81: 243].‘Hottentot’ is a derogatory term of reference to the Khoikhoi or San people, [153: 414, 40 (e), 197] or ‘an offensive mode of address to a coloured person’ [81: 243]. In *CEPPWAWU obo Van Wyk v Atlantic Oil*, the employee referred to ‘coloured’ individuals as ‘hotties’ [26: Du Plessis JS [28]]. The decision-maker found that ‘the evidence shows that racist remarks, such as …[‘h]otties’ when referring to people of colour was racist in its context’ [26: Du Plessis JS [55]].

The ‘k-word,’ ‘known most pointedly for its license of violence towards Blacks during apartheid’^39^ has been described as the ‘most offensive word that can be used towards a black person in South Africa’ [81: 280]. It was used during the colonial and apartheid eras to ‘denigrate,’‘dehumanise’^40^ and treat black bodies as ‘non’ or ‘sub’ human and is still viewed as ‘taboo’ due to its racist connotations [123: 54, 34: 43–44]. It is the ‘highly offensive’ [34: 43], ‘contemptuous,’ ‘abusive’ ‘racial insult’ for Black African individuals [153: 45]. It causes ‘humiliation’^41^ and is viewed as ‘unpardonably painful and violent.’^42^ Despite the use of the word leading to fines and incarceration for *crimen iniuria*,^43^ the use of the hateful term circulates in the South African landscape. The word was used in 7% (8/120) of the sample, despite various decision-makers repeatedly noting that the use of the ‘k–word’ was ‘unacceptable even as jokes.’^44^

Like with justifications for simian metaphors, employees in the sample tended to attempt to downplay their use of the ‘k-word’, as humour. In *Kganu v Smollan Group* the employee stated that he was ‘just sending a laughable picture amongst his colleagues…’ and believed that the use of the ‘k-word’ word as ‘a jokeable and a laughable term which can be used willy-nilly’(sic) [91: Mookamedi NB [43]–[44]]. Similarly, the employee in *Weitz v Southern Mapping Company* denied that he was racist but admitted that the joke contained a racist remark with reference to the ‘k-word’ [227: Zwane TR [14], [27]]. The decision-maker found that the online ‘jokes’ were ‘steeped in race’ [227: Zwane TR [28]]. Likewise, the employee who referred to a ‘self-made’ motorcycle driven by black individuals as a ‘K****saki’ in *Wrobel v Southern Cape Business Systems* justified it as ‘funny and not racist’ [234: du Plessis JS [14]]. The decision-maker noted the material had a ‘racially offensive element’ in which the ‘k-word’ was used [234: du Plessis JS [44]]. According to Pérez, ‘race-based amusement and humour’ continues to have a significant role in contemporary ‘racist discourse’ by feeding ‘racist sentiments and ideologies’ guised as ‘jokes’ [156: 957]. In this sense, the ‘dark side’ of humour is used to divide social groups, alienating the ‘othered,’ and perpetuates notions of racial superiority and inferiority [156: 957].

In *McCarthy v Intergritron*, the employee who used the ‘k word’ in his message said the meaning of the ‘k word’ is ‘non-believer’ [124: Mashigo T [12]]. Similarly, the employee in *Mahri v Mather Dangor Financial Services* explained that the Arabic meaning of the term ‘k*****’ was ‘a non-believer; an ignorant person who is not of the Muslim faith,’ and that it was not ‘racist’ nor ‘derogatory’ [112: Frohnapfel B [15]–[16]]. The decision-maker in *Mahri* categorically noted that ‘[a]lthough the term originated in Asia, in colonial and apartheid South Africa it acquired a particularly excruciating bite and a deliberately dehumanising or delegitimising effect’ [112: Frohnapfel B [29]]. He further noted that ‘[i]n South Africa, the term is racially offensive, derogatory and also amounts to hate speech’ [112: Frohnapfel B [30]].

A further reference to black bodies in the sample was the term ‘flatnose’ [100: Edwards G [4.1.7]] and ‘darkie’ [66: Motsoeneng M [114], 161: van der Merwe F [13]]. ‘Darkie,’ in its original form was used in a ‘paternalistic, condescending manner,’ but the contemporary use is ‘mainly to disparage’ [153: 223]. In *Prince v Nestle Mossel Bay,* the reference to ‘darkies’ in the ‘context’ and ‘chronology of the texts’ exchanged between the employees ‘was not simply a reference to people of darker skin, but, in the context, …[c]onfirmation of the “K-word”’ and was deemed ‘equally derogatory and offensive’ [161: van der Merwe F [43]].

In summary, this section identified in the sample the endurance of hateful racialised ethnophaulisms in South Africa. It showed how a complex lexicon of racist name-calling emergent in the colonial and apartheid past continues in South Africa. The next section focuses particularly on apartheid specific signs and symbols.

### Apartheid-Era Signs and Symbols

Images and physical objects such as national monuments and flags can also convey racial hatred. Reichl notes that flags present ‘very powerful symbols’ of ‘national identity:’ ‘an expression of collective experience’ [169: 207]. National symbols, according to Bornman, are contemporary ‘totems’ typifying nation states [11: 184]. Flags which are associated with a particular political, racial or ethnic interest in a heterogenous society will undoubtedly be viewed as ‘divisive’ [49: 5, 36: 222]. As symbols of national identity and unity, these flags then exclude minority peoples and can signify the divisions within a nation [49: 5, 9]. These flags, such as the ‘old’ Union flag of South Africa, are symbols of hate which, according to Whillock, remind the receiver of ‘specific cultural interpretations of significant events’ and therefore have ‘specific contextual meanings’ [229: 124]. They are ‘the threads in the fabric of our collective memory,’ [229: 124] and have ‘emotional and semiotic significance’ [229: 134].

#### Flags

Flags were designed and have been consistently used as ‘the most precise…[f]orms of non-verbal communication’ [115: 133].^45^ The waving of a white flag to signal surrender or defeat [102: 56, 52 (c)]; the yellow flag flown alone on a sea vessel to signal absence of disease and request for pratique [30 (b)] or the display of the rainbow flag as the international symbol of LGBTQ + pride and gay community [74: 553–572, 61]. More specifically, national flags present ‘very powerful,’ ‘pervasive’ symbols of ‘national identity’ [6: 177, 169: 207, 187: 407, 182: 85, 91], ‘nationhood’ and ‘unity’ [115: 156].

National flags are not merely ‘empty vessels’ [49: 11] circulating in a ‘social vacuum’ [37: 519] but are, according to Malan, ‘a living thing…[t]he repository of national sentiment’ [184: 64–65]. Reichl and Sadowski further note that national flags also convey the ‘expression of collective experience;’ [169: 207] the symbolic ‘carriers’ of a ‘collective memory’ [182: 85]. Therefore, when interpreting the semiotics and ‘social life’ of objects [37: 528], the ‘meaning’ of a sign must consider both the signifier that ‘produces’ or ‘encodes it,’ [169: 211, 37: 519] but also the individual that ‘consumes,’ ‘decodes,’ ‘views’ or ‘interprets it’ in the specific context [169: 211, 37: 519, 209: 45]. Accordingly, the ‘spatial’ and ‘temporal’ contexts in which a flag is both presented and perceived is essential to the symbolic power the object occasions [128: 689, 694].

However, national flags do not only present symbols of national ‘unity.’ The symbolism of flag design, colours and colour combinations, particularly in diverse populations, can have the potential of ‘erecting barriers’ and ‘creating divisions’ amongst a nation rather than unifying a people [16: 60]. Flags associated with particular political, racial or ethnic interests in diverse societies may certainly be viewed as ‘divisive’ [49: 5, 36: 222]. As symbols of national identity and unity, these flags then exclude people [49: 5, 110: 90] and can signify the divisions within a nation [49: 7, 96: 679]. Consequently, as ‘social representations,’ flags are one way in which members of society share their heritage and identify with other members of that grouping [36: 215], ‘both past and present’ [39: 1]. Conversely, the same national flag may simultaneously ‘exert similarly powerful centrifugal effects’ [45: 310] evoking ‘strong feelings of oppression’ or ‘hatred’ amongst other individuals in the same society [39: 1].

The ‘reaction’ of a people to a divisive flag, is truly the response to the ‘entity represented’ by that flag [95: 57]. Within South Africa, the ‘old’ national flag is not merely a ‘stick with a rag on it’ [182: 86]. It is, instead, anchored in ‘historical context’ with a ‘cultural significance’ which transcends the physical materiality of cloth and pigment [229: 126]. The ‘Oranje-Blanje-Blou’ [40 (g)]^46^ for a majority South Africans, is symbolic of a racially segregated rather than unified nation. The ‘old’ flag is a ‘symbol of othering and subjugation’ [215] imbued with ‘rich…[e]motional connotations' [49: 5]. It is a ‘pariah symbol of racism and oppression,’ of ‘discrimination’ and ‘disenfranchisement’ [207: 1, 170: 3, 36: 222].

**Fig. 3:**
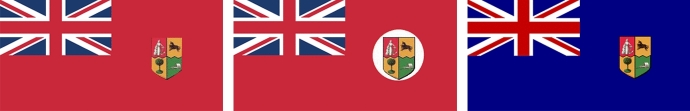
Iterations of the Union of South Africa national flags, 1910 - 1928. www.crwflags.com/fotw/flags/za.html

The ‘current’ South African flag is the third flag of the nation [78: 117]. The Union of South Africa, as a ‘dominion’ of the British Empire, came into being on 31 May 1910 and up until 1928, the ‘Union Jack based ensigns’ were used in the Union as the first flags [16: 43–44, 17: 24–25, 78: 117]. The original Red Ensign had the Union of South Africa’s coat of arms on the ‘field of the fly,’ but as part of the shield was the same colour as the ‘field’ of the ensign, the ‘shield’ was changed to be displayed on a ‘white roundel’ in the fly’ [16: 44, 17: 24–25]. The ‘Red Duster’ was more commonly used than the Blue Ensign [16: 44, 17: 24–25] (See Fig. 3).

**Fig. 4:**
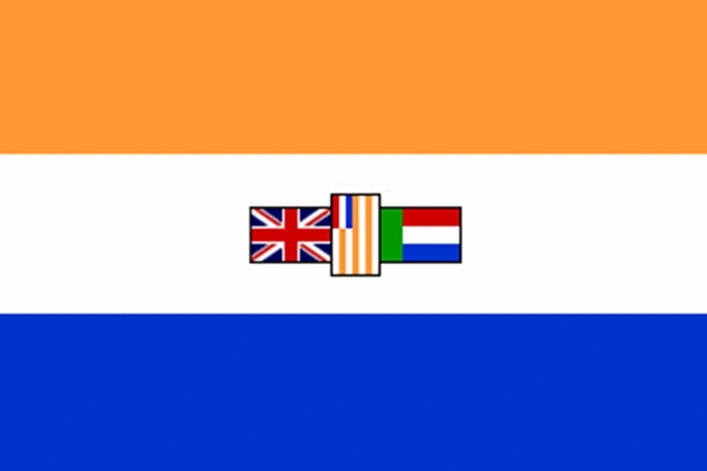
‘Old’ South African flag, 1928 -1994. www.crwflags.com/fotw/flags/za.html

The second flag (which in this paper is referred to as the ‘old’ South African flag) (See Fig. 4) was first hoisted on 31 May 1928 in recognition of the nation’s independence [16: 42]. It represented the European white populace of the country, namely the English-speaking British population and the Afrikaners [11:186]. While the English-speaking population wanted to maintain the Union Jack on the flag, the Afrikaners viewed the Union Jack as a symbol of ‘British domination’ and wished to exclude it entirely from the new national symbol [16: 46, 184: 269–273]. The flag of the Union (commonly known as ‘oranje-blanje-bloue’) as an independent state was comprised of orange-white-blue of the Netherlands as the foundation of the flag (the ‘Prinsenflag’ or ‘Prince’s flag,’ as these were the colours of the Prince of Orange) [15: 61, 16: 48, 109: 146]. This flag was believed to have been the flag hoisted by Van Riebeek upon arrival of the Dutch East India Company in the Cape in 1652 [11: 186, 16: 48, 109: 146]. The positioning the three historical ‘flaglets’ in the center of the white stripe as a singular ‘unit,’ all enjoying equal prominence was regarded, as noted by Bownell, as ‘a heraldic “*tour de force*” probably unique in the history of flags’ [15: 76, 16: 48]. The central white band of the flag comprised the British Union Jack (presenting the British colonies of the Cape and Natal), and the flags of the two Boer Republics, namely the Transvaal ‘Vierkleur’ and the Orange Free State [11: 186, 109: 146].

Bornman notes that while the Union Jack symbolised elements of British colonialism, the ‘old’ Union flag more closely represented the flag of ‘apartheid,’ as it symbolised Afrikaner political pursuits [11: 189].^47^ There was no symbolic representation of the vast majority of South Africa’s Black African, Indian or Coloured inhabitants [15: 103]. The ‘old’ South African flag, according to Maake, represented the ‘symbolic erasure of the indigenous inhabitants of the sub-continent from the landscape of both history and myth’ [109: 145–155]. What further emerges from the sample is that in this sense, the ‘old’ flag presents a means of exerting ‘symbolic dominance and submission;’ keeping ‘racial inferiors’ in their ‘place’ [45: 310–311]. It was therefore for many South Africans, ‘a vivid symbol of white supremacy’ and ‘black disenfranchisement and suppression’ [139: Mojapelo DJP [45]].

In the sample, this was evident in the *Bird v Rand Mutual Admin Services* decision which concerned the dismissal of a white employee for posting a picture of the ‘old’ apartheid era South African flag with the words ‘Good South Africa’ [8: Ngwane N [13]]. The employee’s argument in defence was that the ‘old flag’ was part of his ‘heritage,’ and it had neither been ‘banned’ nor its ‘display forbidden’ (but public display merely ‘discouraged’) [8: Ngwane N [15]]. He did, however, acknowledge that the flag was recognised as a ‘symbol of apartheid’ and ‘took people back to the apartheid era’ [8: Ngwane N [27]]. The employer noted during the proceedings that the ‘old’ South African flag is ‘deemed offensive as it represents the apartheid era, a reminder of the past, the loss of loved ones and injustices of the past’ [8: Ngwane N [17]]. Moreover, it highlighted that the flag is ‘unwelcoming’ to the majority of South Africans [8: Ngwane N [17]]. The decision-maker held that the flag was seen as a ‘symbol of white supremacy over the black majority … [t]hat were oppressed during the apartheid era, a reminder of the past under this flag’ [8: Ngwane N [53]]. The dismissal was held to be the ‘appropriate sanction’ [8: Ngwane N [57]] and the employee’s application disputing the substantive fairness of the decision was dismissed [8: Ngwane N [59]]. It is significant to note that more recently, the South African Equity Court ruled that ‘gratuitous’ displays of the ‘old’ South African flag which do not serve any ‘genuine journalistic, academic or artistic purposes’ amount to prohibited ‘hate speech,’ unfair discrimination and harassment [139: Mojapelo DJP [56], [200], [205]]. However, this decision is currently on Appeal.^48^

#### Racist Images

Images were used in 11% (13/120) of decisions in the sample. The ‘literal,’ ‘linguistic message’ of an image is in the label, caption or text which accompanies the image, while the ‘iconic message’ is the ‘cultural meaning of the symbol’ [229: 126]. The image must have a ‘cultural significance’ for members of a society, as it is that common understanding which infuses the image with a particular meaning [229: 126]. Accordingly, what an image connotes is dependent on the ‘socio-cultural associations’ of the visual community.

In *Maja v Glencore Lion Smelter*, the employee posted an image of ‘white boys riding on the backs of black boys who are on all fours’ with the inscription ‘Vote for DA’ at the top and the words ‘*Kuyobanje*’ which loosely translates as ‘it’s going to be like this’ [113: Talane [6]]. This image was interpreted by the employer to ‘imply’ that voting for the Democratic Alliance (a political party), ‘white people’ would ‘oppress’ or ‘dominate’ black individuals [113: Talane [6], [29]]. The employee maintained that the posts were ‘not intended to hurt anyone or to propagate hatred,’ but were merely ‘intended to encourage people to vote for the ANC’ [113: Talane [65]]. In this instance, the decision-maker found the picture was merely ‘political electioneering’ meant to dissuade Black individuals from voting for the Democratic Alliance, and that ‘there is nothing wrong in that’ [113: Talane [63]].

Similarly, in *Kganu v Smollan Group*, an employee displayed several ‘offensive pictures’ with ‘racial undertones’ and ‘racist remarks’ on social media [91: Mookamedi NB [16], [25], [27]]. One image depicted two groups of chickens. One group appearing in white with the words ‘k****’ and opposite that, the other group of chickens in black with the words ‘*ke mmago*’ as the Sepedi caption, which means ‘k**** is your mother’ [91: Mookamedi NB [17]]. Humour was once again used as a defence, with the employee stating that he was ‘just sending a laughable picture amongst his colleagues,’ and that the ‘k-word’ was ‘a jokeable and a laughable term which can be used willy nilly’ (sic) [91: Mookamedi NB [43]–[44]]. However, under cross-examination, when questioned how he would have felt had his white colleagues posted a similar image with the ‘k-word,’ he responded that he would have felt ‘racially offended’ [91: Mookamedi NB [44]].

In *FAWU obo Myeni v Imperial Retail Solutions*, the employee had posted an image with the caption ‘the official stamp for France on 1912… African Moors beheaded’ [54: Dorasamy AS [8.1]]. The decision-maker noted that ‘there is no doubt that the comments would cause concern as they are not conducive to the transformation of the South African society post the apartheid period,’ further noting that ‘the comments …[were] not only vulgar and leaning towards hate speech but are also offensive towards ‘White people’ [54: Dorasamy AS [11.1]–[11.2]]. These words ‘attacked’ ‘White people generally’ and the ‘derogatory remarks’ were ‘unacceptable’ [54: Dorasamy AS [11.3]]. The decision-maker further noted that the remarks were ‘not only unfair but [were] the subject of debate…[t]o make such remarks and postings on social media a criminal offence’ [54: Dorasamy AS [11.3]]. The comments were found to be ‘offensive’ and not to be in the spirit of nation building in the new South Africa, particularly given the nation’s racially divided past [54: Dorasamy AS [14.5]].

In *Smal v Heineken South Africa* [199: Mokoena K], the employee was dismissed for a series of images which referred to ‘the architect of apartheid,’ former Prime Minister Hendrik Verwoerd and post-apartheid former President Nelson Mandela. The caption on the first image of Mandela reads ‘since 1994 24 206 murders each year’ with a further caption in Afrikaans which translated, stated ‘or is it the angel of death and destruction’ [199: Mokoena K [4.1.2.1]]. The second image was of black bodies hurling stones with Afrikaans text at the bottom of the page which read ‘under the hand of the angel’ [199: Mokoena K [4.1.2.2]]. The third image was one of Hendrik Verwoerd, with a caption above the image which stated, ‘1948–1989, 170 murders each year.’ Next to Verwoerd’s image was a picture of a clean road, apparently in South Africa, with the caption beneath which translated from Afrikaans read ‘under the hand of the devil’ [199: Mokoena K [4.1.2.3]]. The fourth image merely read, ‘dink versigtig en laat jou stem tel’ (think carefully and let your vote count) [199: Mokoena K [4.1.2.4]], while the fifth image is the ‘head’ of Hendrik Verwoerd on the face of a coin from 1967. The caption read ‘when the rand and SA still had value…mooiste munt van alle tye’ (most beautiful mint of all time) [199: Mokoena K [4.1.2.5]]. The final image was a screenshot of an online news headline referencing the South African Human Rights Commission finding in the Equality Court that the Black First Land First organisation was guilty of circulating hate speech.^49^ Beneath this image, translated from Afrikaans, reads ‘do that we will keep fighting until we reach the top again’ (sic) [199: Mokoena K [4.1.2.6]]. The employee stated as her defence that the images she posted were merely aimed at demonstrating that election votes would have a significant impact on whether the country could be improved, which defence was deemed ‘highly improbably and disingenuous’ [199: Mokoena K [5.2]–[5.3]].

In *Mahlangu v Chabo Joubert Air Conditioning Service,* the employee had an image of Robert Mugabe on his profile picture with the caption ‘help the white genocide’ and ‘the only white man you can trust is a dead white man’ [111: Lekgwathi E [13]]. Robert Mugabe was the former President of Zimbabwe, and was ‘one of the leaders of the guerrilla movements against white-minority rule, elected into power in 1980’ [111: Lekgwathi E [13]]. While the employee did not believe there was ‘anything wrong’ in displaying Mugabe’s picture and views on his profile, nor did he find Mugabe’s words offensive, he did under cross examination concede to the ‘sensitivity of the picture’[111: Lekgwathi E [9]]. The employer believed the status was a form of ‘hate speech’ and incited ‘violence against white South Africans’ [111: Lekgwathi E [11]]. The decision-maker found that the image and text was ‘offensive to the white’s minority’ and its aim was ‘achieving the effects of racism through indirect, underhand or divisible means’ (sic) [111: Lekgwathi E [16]]. The words, according to the decision-maker, were ‘clear, unequivocal and overtly racist in nature’ [111: Lekgwathi E [16]]. Moreover, he found that ‘the words speak for themselves’ [16]. The words ‘conjunctively demonstrate the intention to be racist’ and that the profile picture ‘depicts [a] racist remark or a racist slur’ [111: Lekgwathi E [16]]. Similarly, in *Maja v Glencore Lion Smelter*, the employee’s profile picture contained a photo of former President Mugabe and a quotation of the President, ‘South Africans will kick down a statue of a dead white man but won’t even attempt to slap a live one. Yet they can stone to death a black man simply because he is a foreigner’ [113: Talane [5]]. A company witness experienced this image and text to ‘promotes violence against white people as an alternative to black immigrants’ [113: Talane [24]].

From these decisions it can be summarised that both the text and non-textual images circulate hate in online spaces. As an examination of cultural semiotics, the analysis reveals that in South Africa, signs, symbols and signification are culturally and contextually dependant. The sample reveals how animal metaphors and the meanings of certain seemingly banal words within the South African lexicon go beyond the literal, and are symbolically imbued with polysemantic meaning.

## Conclusion

In summary, this paper argued, through a content analysis of 120 first instance South African employment decisions, that employees utilise social media to circulate racialised hate using words and signs with cultural signification or historical connotations. What emerged from the analysis was that hate, whether through words, signs or symbols, circulated between all cultural actors. The analysis specifically highlighted that the use of animal metaphors, words and ethnophaulisms extend beyond their literal meanings. They are decoded and deciphered into referents of textual and non-textual ‘hate’ in digital spaces within the South African employment law context.

## Footnotes

1 ‘Simunye’ is the isiZulu word meaning ‘We are one!’.

2 ‘Rainbow Nation’ is the term coined by the late Archbishop Desmond Tutu to describe post-apartheid South Africa. See Bornman [11: 182].

3 *Constitution of the Republic of South Africa* [31], Chapter 2 Bill of Rights s 16(1) Freedom of expression: ‘Everyone has the right to freedom of expression, which includes (a) freedom of the press and other media; (b) freedom to receive or impart information or ideas; (c) freedom of artistic creativity; and (d) academic freedom and freedom of scientific research.’.

4 *Constitution of the Republic of South Africa* [31], Chapter 2 Bill of Rights s 16(2): ‘The right in subsection (1) does not extend to (a) propaganda for war; (b) incitement of imminent violence; or (c) advocacy of hatred that is based on race, ethnicity, gender or religion, and that constitutes incitement to cause harm.’ See also Mchangama and Alkiviadou [125: 562–563]; Mchangama [126: 335–336].

5 *Promotion of Equality and Prevention of Unfair Discrimination Act* 4 of 2000 (PEPUDA) [163], s 10(1) provides: ‘No person may publish, propagate, advocate or communicate words based on one or more of the prohibited grounds, against any person, that could reasonably be construed to demonstrate a clear intention to (a) be hurtful; (b) be harmful or to incite harm; (c) promote or propagate hatred.’ However, note that the ‘hate speech’ prohibition has been deemed unconstitutional in the Supreme Court of Appeal decision of *Qwelane v South African Human Rights Commission* [165]. The court found the provision overbroad and infringed the constitutionally entrenched freedom of expression. The constitutional invalidity of s 10(1)(a) was confirmed in *Qwelane v South African Human Rights Commission* [166] which declared s 10(1) of the *Promotion of Equality and Prevention of Unfair Discrimination Act* 4 of 2000 ‘unconstitutional and invalid to the extent that it includes the word “hurtful” in the prohibition against hate speech’ as it is inconsistent with s 16 of the Bill of Rights, Chapter 2 of the *Constitution of the Republic of South Africa* [31]*.* The term “hurtful” was deemed ‘vague’ and ‘breached the rule of law’ [166: Majiedt, J [156]]. The Court found that the s 10(1)(a) was an unjustified limitation of the right to freedom of expression enshrined in the Constitution [166: Majiedt, J [198]]. See also Geldenhuys and Kelly-Louw [58: 3], Mchangama and Alkiviadou [125: 563–564; 570–574].

6 The *Promotion of Equality and the Prevention of Unfair Discrimination Act* 2000 (PEPUDA) [163] applies to ‘hate speech’ outside the employment context, while the *Employment Equity Act* [47] applies to those utterances in the workplace. The *Employment Equity Act* [47] does not define ‘hate speech,’ but prohibits ‘unfair discrimination’ based on ‘race’ in s 6. Geldenhuys and Kelly-Louw note that ‘racial slurs’ in the employment context would qualify as ‘hate speech’ as racial slurs ‘advocate hatred based on race’ [58: 8].

7 The *Prevention and Combating of Hate Crimes and Hate Speech Bill* [160] is currently pending. In terms of the Bill, s 4(1) (a) ‘any person who intentionally publishes, propagates or advocates *anything* or communicates to *one or more persons* in a manner that could reasonably be construed to demonstrate a clear intention to be harmful or to incite harm; or promote or propagate hatred’ based on any one or more of a list of grounds is guilty of the offence of hate speech (own emphasis added). The definition of ‘communication’ in terms of s 1 of the Bill includes any ‘display; written, illustrated, visual or other descriptive matter; oral statement; representation or reference; or an electronic communication, and ‘electronic communication’ means ‘a communication by means of data messages.’ ‘Data’ means ‘electronic representations of information in any form’, while ‘data message’ is means ‘data generated, sent, received, or stored by electronic means’ (s 1). Therefore, as the definition of hate speech in the Bill is very broad, it would include messages or posts on social media sent to at least one person under s 4(1)(a); or distributed or shared in terms of s 4(1)(b). See also Rajuili and Nyathi [167: 48]; Mchangama and Alkiviadou [125: 563]; Mchangama [126: 336].

8 For example, under the *International Covenant on Civil and Political Rights* [84] Article 19 reads: ‘Everyone shall have the right to freedom of expression; this right shall include freedom to seek, receive and impart information and ideas of all kinds, regardless of frontiers, either orally, in writing or in print, in the form of art, or through any other media of his choice. Article 20 reads: (1) ‘any propaganda for war shall be prohibited by law; (2) any advocacy of national, racial or religious hatred that constitutes incitement to discrimination, hostility or violence shall be prohibited by law.’.

9 Including whether ‘the employee contravened a rule regulating conduct in the workplace and if a rule was contravened, whether or not the rule was a valid or reasonable. Where the rule was contravened, was the employee aware, or could reasonably be expected to have been aware of the rule; was the rule consistently applied by the employer and lastly, was dismissal an appropriate sanction for the contravention of the rule.’ Schedule 8 Item 7 of the *Labour Relations Act* 1995 [98] as amended.

10 Bargain councils are established in terms of s27 of the *Labour Relations Act* [98: s 27]. Budlender and Sadeck [20: 5] note that ‘bargaining councils are established when employer and employee bodies (unions) in a particular industrial sector and geographical area agree to come together to engage in collective bargaining.’ In terms of s28 of the *Labour Relations Act*, the powers and functions of bargaining councils include the prevention and resolution of labour disputes [98: s 28].

11 For example, Tokiso Dispute Settlement (Pty) Ltd (1 Dec 2019 to 30 November 2022).

12 *Labour Relations Act* 1995 [98] s 143 (1) reads: ‘An arbitration award issued by a commissioner is final and binding and it may be enforced as if it were an order of the Labour Court in respect of which a writ has been issued.’ s 143(5) states: ‘Despite subsection (1), an arbitration award in terms of which a party is required to pay an amount of money must be treated for the purpose of enforcing or executing that award as if it were an order of the Magistrate’s Court.’.

13 This is 68.2% of the total South African population as at 1 January 2022 [90: 16].

14 The worldwide average is significantly lower at 06h58 at January 2022 [Global Digital 2022: 18, 27].

15 The worldwide average is significantly lower at 02h27 at January 2022 [Global Digital 2022: 18, 94].

16 As at 1 January 2022, there were 108.6 million mobile connections in South Africa, which is the equivalent to 179.8% of the total South African population in January 2022 [90: 16].

17 See further eg, *CEPPWAWU obo Van Wykv Atlantic Oil* [26: Du Plessis JS [56]], *Ntshangase v MCFI International SA* [143: Jazbay SA [23]], *SACCAWU obo Tshoeuv Kievitskroon Country Estate* [181: du Plessis L [11]], *Pieters v Southern Canned Products* [158: Goldman B [9]], *Ndhlovu v AdveTech Copperleaf College* [138: Sithole LA [11]].

18 See, eg, *Population Registration Act* [159] which required individuals to be classified from birth as a particular racial group, and an individual’s identity number reflected their race. In terms of the definition of ‘white person:’ means a person who in appearance obviously is, or who is generally accepted as a white person, but does not include a person who, although in appearance obviously a white person, is generally accepted as a coloured person (see s1(xv) of the Act).

19 See, eg, *Constitution of the Republic of South Africa* [31], *Promotion of Equality and the Prevention of Unfair Discrimination Act* [163], *Employment Equity Act* [47].

20 See, eg, *Chiloane v Trans Africa Projects* [27: Kona T [9]], *Ndhlovu v AdveTech Copperleaf College* [138: Sithole LA [11]], *Pieters v Southern Canned Products* [158: Goldman B [9]], *Ackerman v CCI Call Centres* [1: [5.5.2], [6.1.6]].

21 See, eg, *Shivambu v Afripol Security* [193: Hlaba N [4.9]].

22 See more recently *Qwelane* [166: Majiedt J [67]–[77]].

23 See also *Somo v LSC Hospitality* [204: Mathebula S [45], *Ngoepe v Quemic* [141: Mooi F [25]].

24 See e.g., *Hoskins v Standard Insurance Limited* [79*:* Deysel A], *Cantamessa v Edcon Group* [23: Khumalo B]. The words used are found in the Labour Court case *Edcon Limited v Cantamessa* [46: Cele J [3]]: “Watching Carte Blanch and listening to these fucking stupid monkeys running our country and how everyone makes excuses for that stupid man we have to call a president… President my fucking ass!! #zumamustfall This makes me crazy ass mad” (sic).

25 See e.g., *Jikela* [87: Phillips], *Ntshangase v MCFI International* [143: Jazbay SA], *Kalik* [89: Mlaba N].

26 See Hund [83: 239–240]: “Volk” was also used in German meaning ‘no discreet race but a racial mixture comprising components of different value (with the ‘Nordic race’ at the top)’.

27 See *Turner v Welridge Academy* [219: Myhill E [4]] wherein the employee in a constructive dismissal case posted on Facebook ‘If he wants loyalty from his staff, he’ll buy a dog!!’.

28 See, eg, *Shozi v Standard Bank* [194: Lyster R [5]], *Ntshangase v MCFI International SA* [143: Jazbay SA [18]], *Watson v SBV Services (Pty) Ltd* [226: Dhlodhlo P [30]], *Ngeniswa v Fidelity Security Services* [140: Bennett CM [10]] (reference made after dismissal)].

29 The *Oxford Learner’s Dictionary* [149] defines ‘bitch’ as ‘a female dog.’ See also Kleinman, Ezzell and Frost [94: 58].

30 The term coined by phycologist A.A Roback in 1954. See also Hughes [81: 221].

31 Bornman [11: 184] similarly notes that South Africa has ‘9 Black language groups associated with distinct Black ethnic identities…with strong ties to certain regions’ for example the Zulu nation regard Kwa-Zulu-Natal as their nation’s ‘homeland.’

32 The word ‘rooinek’ literally means ‘red-neck.’ It is an epithet for ‘Englishman’ because the Englishman coming to South Africa was unaccustomed to the hot, glaring sunshine, usually burnt red.

33 ‘Soutpiel’ or ‘soutie’ means the British had one foot in South Africa and one foot in Britain—another extremity dangled in the salt waters between the continents.

34 ‘Baas’ (Afrikaans, from Dutch) which means ‘boss’ or ‘master’ and ‘used especially by nonwhites when speaking to or about Europeans in positions of authority’ [129 (b)].

35 *CEPPWAWU obo Van Wyk, D v Atlantic Oil* [26: Du Plessis JS [7(iv)]], *EAMWUSA obo van Duncan Wyk v Dart Stationers* [44: Madotyeni Z [17]].

36 However, see *Duncanmec v Gaylard* [43: [37]] in which case the decision-maker held that ‘boer’ is not racially offensive. See further Geldenhuys and Kelly Louw [58].

37 See, eg, *Mosala v Fidelity Security Services* [131: Basholo V [4.1.4]].

38 In this instance, ‘Kill the Boer’ is seemingly a reference to the symbolic political struggle song against apartheid ‘Dubul’ibhunu’ (translates to Shoot the Boer). See Erlmann [50: 274–301] and Mchangama and Alkiviadou [125: 574–575] for a discussion on the *AfriForum v Malema* (2011) Equity Court decision that the song was used to ‘dehumanise’ the ‘enemy’ (see [62] of the decision).

39 *SARS v CCMA* [205: [38]] in *Mahri v Mather Dangor Financial Services* [112: Frohnapfel B [3]].

40 *Prinsloo v The State* [162: [20]] in *Mahri v Mather Dangor Financial Services* [112: Frohnapfel B [6]].

41 *S v Puluza* [177] quoted with approval in *Ryan v Petrus* [174] in *Mahri v Mather Dangor Financial Services* [112: Frohnapfel B [3]].

42 *SARS v CCMA* [205: [38]] in *Mahri v Mather Dangor Financial Services* [112: Frohnapfel B [3]].

43 See, eg, *Nkondo & SAHRC v Momberg* [28] and *S v Mombe*rg [176] wherein the South Gauteng High Court confirmed the conviction and sentence imposed.

44 See, eg, *Solidarity obo Govender v Air Traffic Navigation Services* [202: Mohlala M [37]], *SACCAWU obo Mokhothu v Edcon* [180: Basholo V [10]], *UASA obo Prinsloo v South Deep Gold Mine* [220: Shear L [4]].

45 See also Erokhina and Soboleva [51: 333–352].

46 The colours of the former national flag; the title of a patriotic Afrikaans song.

47 See also Brownell [15: 103] and Brownell [16: 49].

48 This case is currently on Appeal in the Supreme Court of Appeals. See Mchangama and Alkiviadou [125: 575–576] for a discussion on *Nelson Mandela Foundation Trust* [139: Mojapelo DJP]. However, see de Villiers [36] on *Nelson Mandela Foundation Trust* [139].

49 This is likely a reference to the case *Strydom v Black First Land First* [2019] ZAEQC 1 [208] and the BFL slogan, ‘Land or Death.’ See media statement by the South African Human Rights Commission [206].

## References

[1] *Ackerman v CCI Call Centres (Pty) Ltd*, KNDB9016-19.

[2] *Alexander v Ebesa Architects (Pty) Ltd*, WECT19446-1.

[3] *ANC v Sparrow* [2016] ZAEQC 2016.

[4] Ashforth Adam, Nattrass Nicoli (2005). Ambiguities of ‘Culture’ and the Antiretroviral Rollout in South Africa. Social Dynamics.

[5] Baderoon Gabeba (2017). Animal likenesses, dogs and the boundary of the human in South Africa. Journal of African Cultural Studies.

[6] Bankov, Kristian. 2021.Flags, Identity, Memory: From Nationalisms to the Post-truth Uses of Collective Symbols. In *Flags, Color, and the Legal Narrative*: *Public Memory, Identity, and Critique*, ed. Anne Wagner and Sarah Marusek, 173–190. Springer.

[7] Billig, Michael. 2005. Comic Racism and Violence. In *Beyond a Joke : The Limits of Humour*, ed. Sharon Lockyer and Michael Pickering, 25–44. Palgrave Macmillan.

[8] *Bird v Rand Mutual Admin*, GAJB 15348-17.

[9] Black, Max.1962. *Models and Metaphors*. *Studies in Language and Philosophy*. Cornell University Press.

[10] *Booysen v Namaqua Wines SA (Pty) Ltd,* WECT24746-19.

[11] Bornman, Elirea. 2014. Post-apartheid South Africa: A United or a Divided Nation? In *Symbols that Bind, Symbols that Divide. The Semiotics of Peace and Conflict,* ed. Scott L. Moeschberger, Rebekah A. Phillips DeZalia, 181–205. Springer.

[12] Botha MM (2018). Managing Racism in the Workplace. Journal for Contemporary Roman-Dutch Law.

[13] Bowker, Geoffrey and Susan Leigh Star. 1999. *Sorting Things Out: Classification and Its Consequences.* MIT Press.

[14] Brons Lajos (2015). Othering, An Analysis. Transcience: A Journal of Global Studies.

[15] Brownell, Fredrick. 2015. Convergence and Unification: The National Flag of South Africa (1994) in Historical Perspective. (Doctor Philosophiae Thesis, University of Pretoria, 2015).

[16] Brownell Frederick Gordon (2011). Flagging the “new” South Africa, 1910–2010. Historia.

[17] Brownell, Frederick Gordon. 1993. *National and flora and fauna emblems of the Republic of South Africa.* C. van Rensburg Publications.

[18] Bruneau Emile, Kteily Nour (2017). The enemy as animal: Symmetric dehumanization during asymmetric warfare. PLoS ONE.

[19] Buckland Adam, Nattrass Nicoli (2020). Understanding Preferences for Humane and Cruel Treatment of Pest Rodents in Site C, Khayelitsha, South Africa. Journal of Applied Animal Welfare Science.

[20] Budlender, Debbie and Shaheeda Sadeck. 2007. Bargaining Councils and other benefit schemes. Report for National Treasury, Community Agency for Social Enquiry. http://www.treasury.gov.za/publications/other/ssrr/session%20one%20papers/budlender%20barg%20coun%20funds%20analysis.pdf.

[21] Burchell Jonathan (2019). Balancing equality of respect with freedom of expression: The *actio iniuriarum* and hate speech. Acta Juridica.

[22] Cambridge Academic Content Dictionary, Cambridge University Press. ‘rubbish’ accessed 1 April 2022. https://dictionary.cambridge.org/dictionary/english/rubbish.

[23] *Cantamessa v Edcon Group*, GAJB5735-16.

[24] Carroll Noël (2020). I’m Only Kidding: On Racist and Ethnic Jokes. The Southern Journal of Philosophy.

[25] Cassim F (2015). Regulating Hate Speech and Freedom of Expression on the Internet: Promoting Tolerance and Diversity. South African Journal of Criminal Justice.

[26] *CEPPWAWU obo Van Wyk, D v Atlantic Oil*, WEGE2685-16.

[27] *Chiloane v Trans Africa Projects (Pty) Ltd*, GATW14881-20.

[28] *Clement David Nkondo and The South African Human Rights Commission v Vicky Momberg* Case No EQ007/201.

[29] *Code of Good Practice: Dismissal* Schedule 8 of the *Labour Relations Act* 1995.

[30] *Collins English Dictionary.* (2022). (a) ‘bitch.’ Accessed 31 March 2022 from https://www.collinsdictionary.com/dictionary/english/bitch. (b) ‘yellow flag’ and ‘quarantine flag.’ Accessed 31 March 2022 from https://www.collinsdictionary.com/dictionary/english/yellow-flag.

[31] *Constitution of the Republic of South Africa* 1996 (South Africa).

[32] Cornish René, Tranter Kieran (2019). The Cultural, Economic and Technical Milieu of Social Media Misconduct Dismissals in Australia and South Africa. Law in Context.

[33] Crary, Alice. 2021. Dehuminization and the Question of Animals. In *The Routledge Handbook of Dehumanization*, ed. Maria Kronfeldner, 159–172. Routledge.

[34] de Klerk Vivian Anne (2011). A nigger in the woodpile? A racist incident on a South African University campus. Journal of Languages and Culture.

[35] de Robillard Benita, Lipschitz Ruth (2017). Race and “the Animal” in the Post-Apartheid ‘National Symbolic. Image and Text.

[36] de Villiers, Bertus (2022). *Navigating the Unknown: Essays on Selected Case Studies about the Rights of Minorities*. Brill.

[37] Del Percio, Alfonso. 2015. On the social life of a city anthem: Semiotic objects, ideologies of belonging, and the reproduction of sociocultural difference. *Social Semiotics* 25 (4): 517–531.

[38] Delgado Richard, Stefanic Jean (2004). Understanding Words that Wound.

[39] DeZalia, Rebekah and Scott Moeschberger. 2014. The Function of Symbols that Bind and Divide. In *Symbols that Bind, Symbols that Divide. The Semiotics of Peace and Conflict,* ed. Rebekah DeZalia and Scott Moeschberger, 1 -12. Springer.

[40] *Dictionary of South African English*. (2022). (a) ‘Baas,’ n. Accessed 02 February 2022 from https://dsae.co.za/entry/baas/e00443. (b) ‘Coolie,' n. Accessed 02 February 2022 from https://dsae.co.za/entry/coolie/e01794. (c) ‘Crunchie,' n. Accessed 02 February 2022 from https://dsae.co.za/entry/crunchie/e01854. (d) ‘Dutchman,' n. Accessed 02 February 2022 from https://dsae.co.za/entry/dutchman/e02182. (e) ‘Hottie,' n. Accessed 01 February 2022 from https://dsae.co.za/entry/hottie/e03110. (f) ‘Inja,' n. Accessed 01 February 2022 from https://dsae.co.za/entry/inja/e03288. (g) ‘Oranje-Blanje-Blou.’ Accessed 06 March 2022 from https://m.dsae.co.za/#!/word/5397/orange-blanje-blou. (h) ‘Rockspider,' n. or ‘rock,' n. Accessed 02 February 2022 from https://dsae.co.za/entry/rockspider/e06040 and https://dsae.co.za/entry/rock/e06033. (i) ‘Rooinek,' n. Accessed 06 March 2022 from https://dsae.co.za/entry/rooinek/e06097#:~:text=Origin%3A,%3B%20redneck%3B%20rooineck%3B%20soutie. (j) ‘Soutpiel,' n. or ‘Soutie,' n. Accessed 06 March 2022 from https://dsae.co.za/entry/soutpiel/e06732.

[41] du Plessis, Pieter. 2019. On ‘dirty’ rats, ‘dirty’ spaces and slow violence in Site C, Khayelitsha: An interdisciplinary ethnography of the everyday living in a rat-infested area. MPhil Thesis, University of Cape Town.

[42] du Toit Louise (2019). The African Animal Other: Decolonizing nature. Angelaki : Journal of Theoretical Humanities.

[43] *Duncanmec (Pty) Limited v Gaylard* 2018 6 SA 335 (CC).

[44] *EAMWUSA obo van Duncan Wyk v Dart Stationers CC*, WECT14283-11.

[45] Eckels, Christopher Wood. 2021.The Antisocial Fabric: German and American Approaches to Flags As Hate Speech in Public Demonstration. In *Flags, Color, and the Legal Narrative*: *Public Memory, Identity, and Critique*, ed. Anne Wagner and Sarah Marusek, 309–332. Springer.

[46] *Edcon Limited v Cantamessa and Others* (JR30/17) [2019] ZALCJHB 273.

[47] *Employment Equity Act* 55 of 1998.

[48] Enwezor Okwui (1997). Reframing the black subject ideology and fantasy in contemporary South African representation. Third Text.

[49] Eriksen, Thomas Hylland. 2007. Some questions about flags. In *Flag, Nation and Symbolism in Europe and America*, ed. Thomas Hylland Eriksen and Richard Jenkins, 1–7. Routledge.

[50] Erlmann Viet (2020). ‘Shoot the Boer’: Hate Speech, Law and the Expediency of Sound. Law Text Culture.

[51] Erokhina, Yulia and Anita Soboleva. 2021.Semiotic and Legal Analysis of Flags in Russia: Belonging to a Multi-National Federal State Through Color, Form, Space and Time. In *Flags, Color, and the Legal Narrative:Public Memory, Identity, and Critique*, ed. Anne Wagner and Sarah Marusek, 333–352. Springer.

[52] *Farlex Dictionary of Idioms.* (2015). (a) ‘tail between legs’ (n.d.) Accessed 9 March 2022 from https://idioms.thefreedictionary.com/tail+between+legs. (b) ‘to rat on someone’ (n.d.) Accessed 6 April 2022 from https://idioms.thefreedictionary.com/rat+on+someone. (c) ‘wave a white flag’ Accessed 16 March 2022 from https://idioms.thefreedictionary.com/wave+a+white+flag.

[53] *FAWU obo Du Preez, Marlin v Aanhalt Boerdery*, WEGE1417-13.

[54] *FAWU obo Myeni, Ntokozo v Imperial Retail Solutions*, KNDB2662-16.

[55] *FAWU obo Ngcangisa, N v Premier FMCG (Pty) Ltd,* WECT4691-19.

[56] Felmlee Diane, Rodis Paulina Inara, Zhang Amy (2020). Sexist Slurs: Reinforcing Feminine Stereotypes Online. Sex Roles.

[57] Geldenhuys Judith, Kelly-Louw Michelle (2020). Demystifying Hate Speech under PEPUDA. Potchefstroom Electronic Law Journal.

[58] Geldenhuys Judith, Kelly-Louw Michelle (2020). Hate speech and racist slurs in the South African context: Where to start?. Potchefstroom Electronic Law Journal.

[59] Goatly Andrew (2006). Humans, Animals and Metaphors. Society and Animals.

[60] Goff Phillip (2008). Not yet human: Implicit knowledge, historical dehumanization, and contemporary consequences. Journal of Personality and Social Psychology.

[61] Gonzalez, Nora. How Did the Rainbow Flag Become a Symbol of LGBTQ Pride?’ *Encyclopedia Britannica* https://www.britannica.com/story/how-did-the-rainbow-flag-become-a-symbol-of-lgbt-pride. Accessed 17 March 2022.

[62] Green Louise (2016). Apartheid’s wolves: Political animals and animal politics. Critical African Studies.

[63] Green Lesley (2020). Rock Water Life.

[64] Grogan, J. 2017. *Dismissal*. Juta.

[65] Grove Natalie, Zwi Anthony (2006). Our health and theirs: Forced migration, othering, and public health. Social Science and Medicine.

[66] *Gumede v Mutual & Federal*, GAJB9817-14.

[67] Harmer, Emily and Karen Lumsden. 2019. Online Othering: An Introduction. In *Online Othering Exploring Digital Violence and Discrimination on the Web*. *Palgrave Studies in Cybercrime and Cybersecurity*, ed. Karen Lumsden and Emily Harmer, 1–33. Cham: Palgrave Macmillan.

[68] *Harvey v Little Gems Residential & Respite Care*, GAJB9921-17.

[69] Haslam, Nick, Elise Holland and Michelle Stratemeyer. 2019. Kittens, pigs, rats, and apes: The psychology of animal metaphors. In *Why We Love and Exploit Animals: Bridging Insights from Academia and Advocacy,* ed. Gordon Hodson and Kristof Dhont, 90–103. Routledge.

[70] Haslam Nick, Loughnan Stephen, Sun Pamela (2011). Beastly: What Makes Animal Metaphors Offensive?. Journal of Language and Social Psychology.

[71] Hanslam Nick, Kronfeldner Maria (2021). The Social Psychology of Dehumanization. The Routledge Handbook of Dehumanization.

[72] Haslam Nick (2006). Dehumanization: An Integrative Review. Personality and Social Psychology Review.

[73] Hate Speech Information Sheet at https://www.sahrc.org.za/home/21/files/Hate%20Speech%20Information%20Sheet-%20print%20ready-.pdf.

[74] Hauksson-Tresch, Nathalie. The Rainbow Flag as Signal, Icon, Index and Symbol of Collective and Individual Gay Identity. In *Flags, Color, and the Legal Narrative: Public memory, identity, and critique,* ed. Anne Wagner and Sarah Marusek, (Springer, 2021) 553–572.

[75] Heryono Henri, Helmy Faisal (2018). Semiotics at Discursive Texts and Images in Online News. International Journal of Engineering & Technology.

[76] Hodson Gordon, Kteily Nour, Hoffarth Mark (2014). Of Filthy Pigs and Subhuman Mongrels: Dehumanization, Disgust, and Intergroup Prejudice. Testing, Psychometrics, Methodology in Applied Psychology.

[77] Hodson Gordon, Rush Jonathan, MacInnis Cara (2010). A “joke is just a joke” (except when it isn’t): Cavalier humor beliefs facilitate the expression of group dominance motives. Journal of Personality and Social Psychology.

[78] Horvath, George. 2018.The Semiotics of Flags: The New Zealand Flag Debate Deconstructed. In *Language and Literature in a Glocal World,* ed. Sandhya Rao Mehta, 115–126. Oman: Springer.

[79] *Hoskins v Standard Insurance Limited*, KNDB11934-17.

[80] Howitt, Dennis and Kwame Owusu-Bempah. 2005. Race and Ethnicity in Popular Humour. In *Beyond a Joke : The Limits of Humour*, ed. Sharon Lockyer and Michael Pickering, 45–62. Palgrave Macmillan.

[81] Hughes Geoffrey (2006). An Encyclopedia of Swearing : The Social History of Oaths, Profanity, Foul Language, and Ethnic Slurs in the English-Speaking World.

[82] Hund, Wulf, Charles Mills and Silvia Sebastion. 2015. Editorial. In *Simianization: Apes, Gender, Class, and Race,* ed. Wulf Hund, Charles Mills and Silvia Sebastion, 7–16. (LIT Verlag Münster, 2015).

[83] Hund Wulf, Kronfeldner Maria (2021). Dehumanization and Social Death as Fundamentals of Racism. The Routledge Handbook of Dehumanization.

[84] *International Covenant on Civil and Political Rights* (adopted 16 December 1966 by General Assembly Resolution 2200A (XXI).

[85] Jahoda Gustav (1999). Images of Savages.

[86] Jeewa, Tanveer Rashid and Jatheen Bhima, ‘Discriminatory Language: A Remnant of Colonial Oppression’ (2021) 11(1) *Constitutional Court Review* 323, 323–339.

[87] *Jikela v Smit Amandla Marine*, WECT16547-12.

[88] Jolly Pieter (2002). Therianthropes in San Rock Art. The South African Archaeological Bulletin.

[89] *Kalik v Woolworths (Pty) Ltd*, KNDB3821-16.

[90] Kemp, S. (2022). Digital 2022: South Africa at https://datareportal.com/reports/digital-2022-south-africa, Global Digital 2022: https://datareportal.com/reports/digital-2022-global-overview-report.

[91] *Kganu v Smollan Group*, LP8741-16.

[92] Khumalo B (2018). Racism in the workplace : A view from the jurisprudence of courts in the past decade. SA Mercantile Law Journal.

[93] *Khumalo & Others v Holomisa* 2002 (5) SA 401 (CC), 2002 (8) BCLR 771 (CC).

[94] Kleinman Sherryl, Ezzell Matthew, CoreyFrost A (2009). Reclaiming Critical Analysis: The Social Harms of “Bitch”. Sociological Analysis.

[95] Knowlton Steven (2012). Applying Sebeok’s Typology of Signs to the Study of Flags. Raven; A Journal of Vexillology.

[96] Kolsto Pål (2006). National symbols as signs of unity and division. Ethnic and Racial Studies.

[97] Kteily Nour, Bruneau Emile (2017). Darker Demons of Our Nature: The Need to (Re)Focus Attention on Blatant Forms of Dehumanization. Current Directions in Psychological Science.

[98] *Labour Relations Act* 66 of 1995.

[99] Lawrence, Elizabeth Atwood. 1993. The Sacred Bee, the Filthy Pig, and the Bat Out of Hell: Animal Symbolism as Cognitive Biophilia. In *The Biophilia hypothesis,* ed. Stephen Kellert and Edward Wilson, 301 – 341. Island Press.

[100] *Leach v Ipop Suzuki*, MINT44570.

[101] *Leih v World Net Logistics(PTY)LTD*, GAEK8279-17.

[102] Leone, Massimo. 2021. The Semiotics of Flags. In *Flags, Color, and the Legal Narrative*: *Public Memory, Identity, and Critique*, ed. Anne Wagner and Sarah Marusek, 53 – 64. Springer.

[103] *Le Roux and others v Dey* (CCT 45/10) [2011] ZACC 4; 2011 (3) SA 274 (CC); 2011 (6) BCLR 577 (CC).

[104] Lipschitz, Ruth. 2019. Abjection. In *Edinburgh Companion to Animal Studies*, ed. Lynn Turner, Undine Sellbach and Ron Broglio, 13–29. Edinburgh University Press.

[105] Lobban Richard (1994). Pigs and Their Prohibition. International Journal of Middle East Studies.

[106] Lockyer Sharon, Pickering Michael (2008). You Must Be Joking: The Sociological Critique of Humour and Comic Media. Sociology Compass.

[107] Loughnan Steve, Haslam Nick, Kashima Yoshihisa (2009). Understanding the Relationship between Attribute-Based and Metaphor-Based Dehumanization. Group Processes & Intergroup Relations.

[108] *Lucas v Peterson* (EC02/2013) [2016] <<ZANCHC>> 43 (13 December 2016).

[109] Maake, Nhlanhla. 1996. Inscribing Identity on the Landscape: National symbols in South Africa. In *Text, Theory, Space. Land, literature and history in South Africa and Australia,* ed. Kate Darian-Smith, Liz Gunner and Sarah Nuttall, 145 – 155. Routledge.

[110] Mach Zdzislaw (1992). National Symbols in Politics, The Polish Case. Ethnologia Europea.

[111] *Mahlangu v Chabo Joubert Air Conditioning Services*, MEMP259.

[112] *Mahri v Mather Dangor Financial Services*, NWKD2592-18.

[113] *Maja v Glencore Lion Smelter*, LP3649–16

[114] *Makhanya v St Gobain* [2019] 7 BALR 720 (NBCCI).

[115] Makolkin Anna (2001). Flags and flagomania: The visual neoromantic pandemia of the twentieth century. American journal of semiotics.

[116] Maqutu Lindiwe, Motloung Siphiwe (2018). Hidden racial attitudes within the workplace: An evaluation of *Bester v Rustenburg Platinum Mine*. South African Journal on Human Rights.

[117] Marais M (2017). A constitutional perspective on the *Sparrow* judgements. Journal for Juridical Science.

[118] Marschall Sabine (2004). The signifying power of the monumental image. Image and Text : A Journal for Design.

[119] Martin, Amy. 2020. A is for Ape. In *Animalia*. *An Anti-Imperial Bestiary for Our Times* ed. Antoinette Burton and Renisa Mawani, 21–28. Duke University Press.

[120] Marx F (2021). Hate Speech on Social Network sites: Perpetrator and service providers’ liability. Obiter.

[121] *MATUSA obo Elzaan Paulse v JD Kirsten Boerdery (EDMS) BPK*, WECT17777-18.

[122] Mbembe, Achille and Laurent Dubois L. 2017. *Critique of Black Reason.* Duke University Press.

[123] Mbowa Sonia (2020). Exploring the Use of South African Ethnic and Racial Slurs on Social Media. International Journal of Critical Diversity Studies.

[124] *McCarthy v Intergritron*, GAJB26462-17.

[125] Mchangama Jacob, Alkiviadou Natalie (2022). South Africa the Model? A Comparative Analysis of Hate Speech Jurisprudence of South Africa and the European Court of Human Rights. Journal of Free Speech Law.

[126] Mchangama, J. 2022. *Free Speech: A History from Socrates to Social Media.* Basic Books.

[127] *Mduduma v Jabulani Food Lovers Market*, GAJB27114-16.

[128] Medway Dominic (2019). Flags, society and space: Towards a research agenda for vexillgeography. Area.

[129] *Merriam-Webster Dictionary*. (2022). (a) ‘Ape.’ Accessed 28 March 2022 from https://www.merriam-webster.com/dictionary/ape. (b) ‘Baas.’ Accessed 1 February 2022 from https://www.merriam-webster.com/dictionary/baas.

[130] Mondal, Mainack, Leandro Araújo Silva and Fabrício Benevenuto. 2017. A Measurement Study of Hate Speech in Social Media. *Proceedings of the 28th ACM Conference on Hypertext and Social Media.* 85 – 94. <https://doi-org.ezp01.library.qut.edu.au/10.1145/3078714.3078723>.

[131] *Mosala v Fidelity Security Services (Pty) Ltd*, FSBF3982-18.

[132] Mudavanhu Selina (2017). Comrades, Students, Baboons and Criminals: An Analysis of “Othering” on Facebook in Relation to the #Rhodesmustfall/#Feesmustfall Movement at the University of Cape Town. African Journalism Studies.

[133] Mullin Molly (1999). Mirrors and Windows: Sociocultural Studies of Human-Animal Relationships. Annual Review of Anthropology.

[134] *Naidoo v Illovo Sugar S.A Noodsbery*, KNPM3018-18.

[135] *NASECGWU obo Nkomombini, R and Steyn Diamante CC*, NC1038-20.

[136] *Ndaba v Mamba Strike Force*, GAJB132-17.

[137] Ndebele, Njabulo. 2007. *Fine Lines from the Box. Further Thoughts about our Country*. Roggebaai: Umuzi-Random House. Retrieved at <http://www.njabulondebele.co.za/wp-content/uploads/2015/11/The_Year_of_the_Dog_dun.pdf> (online 22 February 2022).

[138] *Ndhlovu v AdveTech Copperleaf College*, GATW12969-18.

[139] *Nelson Mandela Foundation Trust and Another v Afriforum NPC and Others* [2019] 4 All SA 237 (EqC).

[140] *Ngeniswa v Fidelity Security Services (Pty) Ltd*, WECT9843-14.

[141] *Ngoepe v Quemic (Pty) Ltd*, GAJB4016-15.

[142] Nöth Winfried (1990). Handbook of Semiotics.

[143] *Ntshangase v MCFI International SA Pty Ltd*, KNDB10251-15.

[144] Nuessel Frank (2008). A Note on Ethnophaulisms and Hate Speech. Names.

[145] *NUFBWSAW obo Liebenberg, M A v Institute for the Blind*, WECT2668-16.

[146] *NUMSA obo Bheki Ncikazi v Express Employment Professionals*, MIDB18422.

[147] *NUMSA obo Daniels, Comarriyah and 6 others v Polyoak Packaging*, MEWC11858.

[148] Odendal et al. 1992. *Verklarende Handwoordeboek van die Afrikaanse Taal.* Perskor.

[149] *Oxford Learner’s Dictionary* (online). ‘bitch’ is a ‘female dog’ (def 1) from https://www.oxfordlearnersdictionaries.com/definition/american_english/bitch_1. Accessed 21 February 2022.

[150] *Oxford Dictionary of Phrase and Fable* (Oxford University Press, 2006) ‘Hottentot’ accessed 11 March 2022 at <https://www-oxfordreference-com.ezp01.library.qut.edu.au/view/10.1093/oi/authority.20110803095946436>.

[151] Palmatier, Robert. 1995. *Speaking of Animals: A Dictionary of Animal Metaphors.* ABC-CLIO.

[152] Palumbo, Meredith. 2008.The Canine Metaphor in the Visual Arts. In *Canis Africanis : A Dog History of Southern Africa,* ed. Lance van Sittert and Sandra Swart, 263 – 26. BRILL.

[153] Partridge Eric, Dalzell Tom, Victor Terry (2015). The Concise New Partridge Dictionary of Slang and Unconventional English.

[154] Paton, Bernadette. The dog: man’s best friend? In Oxford English Dictionary (Online at 22 February 2022) ‘The dog: man’s best friend?’< https://public.oed.com/blog/word-stories-dog/*>.*

[155] Paustian Megan Cole (2021). Dogs, Whiteness, and the Politics of African Humanity. Modern Fiction Studies.

[156] Pérez Raúl (2017). Racism without Hatred? Racist Humor and the Myth of “Colorblindness”. Sociological Perspectives.

[157] Pickover, Michele. 2005. Animal Rights in South Africa. Juta and Co.

[158] *Pieters v Southern Canned Products*, CHEM362-18~19.

[159] *Population Registration Act* 30 of 1950.

[160] *Prevention and Combating of Hate Crimes and Hate Speech Bill* (B 9 – 2018).

[161] *Prince and 4 Others v Nestle Mossel Bay*, WEGE3011-18.

[162] *Prinsloo v The State* [2014] ZASCA 96 (SCA).

[163] *Promotion of Equality and the Prevention of Unfair Discrimination Act* 4 of 2000.

[164] *PSA obo O'Kelly, S. v SARS - SA Revenue Services*, FSBF109-18.

[165] *Qwelane v South African Human Rights Commission* [2019] ZASCA 167 (Supreme Court of Appeal Judgement).

[166] *Qwelane v South African Human Rights Commission and Another* [2021] ZACC 22 (Constitutional Court Judgement).

[167] Rajuili K, Nyathi Nomagugu (2017). South Africa and Kenya’s Legislative Measures to Prevent Hate Speech. Conflict Trends.

[168] *Rawlins v Southern Suns,* GAJB2324-19.

[169] Reichl Susanne (2004). Flying the Flag: The Intricate Semiotics of National Identity. European journal of English studies.

[170] Resane Kelebogile T (2018). Statues, symbols and signages: Monuments towards socio-political divisions, dominance and patriotism?. Theological Studies.

[171] Rodriguez IL (2009). Of women bitches, chickens and vixens: Animal metaphors for women in English and Spanish. Culture, Language and Representation.

[172] *Roose v Netcare 911*, ECPE405-16.

[173] Roothaan, Angela. 2019. Deconstructing or decolonizing the human–animal divide. In *Indigenous, Modern and Postcolonial Relations to Nature: Negotiating the Environment.* Routledge.

[174] *Ryan v Petrus* 2010 (1) SACR 274 (ECG).

[175] *S v Mamabolo* [2001] ZACC 17; 2001 (3) SA 409 (CC); 2001 (5) BCLR 449 (CC).

[176] *S v Mombe*rg [2019] ZAGPJHC 183, 2019 JDR 1201 (GJ).

[177] *S v Puluza* 1983 (2) PH H150 (E).

[178] Sabinet (Webpage) http://www.sabinet.co.za.

[179] *SACCAWU obo Mmoso, T v Mount Amanzi Holiday Resort*, GATW13332–14.

[180] *SACCAWU obo Mokhothu, Gustav v Edcon (Pty) Ltd*, FSWK824-14.

[181] *SACCAWU obo Tshoeu, Betty v Kievitskroon Country Estate,* GATW8749-19.

[182] Sadowski, Mirosław M. 2021.Fluttering the Past in the Present. The Role of Flags in the Contemporary Society: Law, Politics, Identity and Memory. In Flags, Color, and the Legal Narrative: Public Memory, Identity, and Critique, ed. Anne Wagner and Sarah Marusek, 85–102. Springer.

[183] SAFLII (Webpage) http://www.saflii.org**.**

[184] Saker, Harry. 1977. The South African Flag Controversy, 1925 – 1928 (PhD dissertation, University of Cape Town, 1977).

[185] Salter Phia, Adams Glenn, Perez Michael (2017). Racism in the Structure of Everyday Worlds: A Cultural-Psychological Perspective. Current Directions in Psychological Science.

[186] Saminaden Annick, Loughnan Stephen, Haslam Nick (2010). Afterimages of savages: Implicit associations between primitives, animals and children. British Journal of Social Psychology.

[187] Satherley Nicole, Osborne Danny, Sibley Chris G (2019). Who Is for (or Against) the National Flag? Ideological and Identity-Based Motivators of Attitudes. Analyses of Social Issues and Public Policy.

[188] Sax Boria, Kean Hilda, Howell Phillip (2018). When Adam and Eve were monkeys: Anthropomorphism, zoomorphism, and other ways of looking at animals. The Routledge Companion to Animal-Human History.

[189] *SDTU obo Liebenberg, M A v Mev S Botha Justitute Vir Blundes*, WECT2668-16.

[190] *Shayi v Quality Pourts*, GAJB4751-15.

[191] Shear, Keith. 2008. Police dogs and state rationality in early twentieth-century South Africa*.* In *Canis Africanis: A Dog History of Southern Africa,* ed. Lance van Sittert and Sandra Swart, 193–216. Brill.

[192] Shear, Keith. 2000. Police dogs and state rationality in early twentieth-century South Africa. In *Science and Society in Southern Africa,* ed. Saul Dubow, 143–163. Manchester University Press.

[193] *Shivambu v Afripol Security,* GAJB27234-19.

[194] *Shozi v Standard Bank*, KNDB6956-17.

[195] Sia Sophie, Cornish René, Tranter Kieran (2021). Fired for Facebook…terminated for Tinder: Dismissal for social media misconduct in New Zealand. International Journal of Law and Information Technology.

[196] Siegel, Alexandra. 2020. Online Hate Speech. In *Social Media and Democracy: The State of the Field, Prospects for Reform,* ed. A. Tucker and N. Persily, 56–88. Cambridge University Press.

[197] Silva Penny (1996). Dictionary of South African English on Historical Principles.

[198] Sleep Lyndal, Tranter Kieran (2018). Social media in security decision-making in Australia: An archive of truth?. Media and Arts Law Review.

[199] *Smal v Heineken South Africa (Pty) Ltd*, GAEK7087-19.

[200] Smith, Stephen. 1995. There’s Such a Thing as free Speech: And It’s a Good Thing, Too. In *Hate Speech*, ed. Rita Kirk Whillock and David Slayden. California: Sage Publications.

[201] Smith David, Panaitiu Ioana, Hund Wulf, Mills Charles, Sebastion Silvia (2015). Aping the Human Essence Simianization as Dehumanization. Simianization: Apes, Gender, Class, and Race.

[202] *Solidarity obo Govender, Mark Emslie v Air Traffic Navigation Services*, GAEK2423-18.

[203] Sommer Robert, Sommer Barbara (2011). Zoomorphy: Animal Metaphors for Human Personality. Anthrozoös.

[204] *Somo v LSC Hospitality*, GAJB26014-20.

[205] *South African Revenue Service v Commission for Conciliation, Mediation and Arbitration and Others* [2016] ZACC 38.

[206] South African Human Rights Commission at https://www.sahrc.org.za/index.php/sahrc-media/news-2/item/1907-media-statement-the-south-african-human-rights-commission-welcomes-the-equality-court-s-decision-on-the-blf-and-hate-speech, accessed on 5 April 2022.

[207] Storey, Peter. 2012. Banning the Flag from our churches: Learning from the Church-State struggle in South Africa. In *Between Capital and Cathedral: Essays on Church-State relationships* (Research Institute for Theology and Religion, University of South Africa), ed. Wessel Bentley and Dion Forster. 1–20.

[208] *Strydom v Black First Land First* [2019] ZAEQC 1.

[209] Sturken Marita, Cartwright Lisa (2001). Practices of Looking: An Introduction to Visual Culture.

[210] Suzuki Yuka (2017). The Nature of Whiteness: Race, Animals, and Nation in Zimbabwe.

[211] Swart, Sandra. 2021. African Studies. In *Handbook of Historical Animal Studies,* ed. Mieke Roscher, André Krebber and Brett Mizelle, 85–100. De Gruyter Oldenbourg.

[212] Taljaard-Gilson Gerda (2018). Symbolic values of the dog in Afrikaans literature. Tydskrif vir Letterkunde.

[213] Tambiah Stanley (1969). Animals are good to think and good to prohibit. Ethnology.

[214] Tangye, H. 1896. New South Africa: Travels in the Transvaal and Rhodesia. Horace Cox, cited in Swart, Sandra. 2021. African Studies. In *Handbook of Historical Animal Studies,* ed. Mieke Roscher, André Krebber and Brett Mizelle, 85–100. De Gruyter Oldenbourg.

[215] The Anti-Racism project team, The Institute for Justice and Reconciliation. 2018. ‘Oranje Blanje Blou keeps us anchored in the ugly past’ 16 March 2018. News24 <https://www.news24.com/News24/oranje-blanje-blou-keeps-us-anchored-in-the-ugly-past-20180316>.

[216] Thellefsen, Torkild and Bent Sørensen. 2017. *Umberto Eco in His Own Words.* De Gruyter Mouton.

[217] Thomas, Keith. Man and the Natural World: A History of the Modern Sensibility (Pantheon, 1983). In *Mirrors and Windows: Sociocultural Studies of Human-Animal Relationships*. Molly Mullin. 1999. *Annual Review of Anthropology* 28(1): 201–224.

[218] Tipler Carol, Ruscher Janet (2014). Agency's Role in Dehumanization: Non-human Metaphors of Out-groups. Social and Personality Psychology Compass.

[219] *Turner v Welridge Academy cc*, GAJB6655-15.

[220] *UASA obo Prinsloo, Gert v South Deep Gold Mine - A Division of Gold Fields Ltd*, GAJB22507-16.

[221] *UCIMESHAWU obo Khumalo, Muziwenhlanhla and 1 Other v Gooderson Drakensburg Gardens*, KNPM2894-15.

[222] *Urban Dictionary* definition of ‘dog eater’ from urbandictionary.com at https://www.urbandictionary.com/define.php?term=DOG%20EATER Accessed 21 February 2022.

[223] Vaes Jeroen (2012). We are human, they are not: Driving forces behind outgroup dehumanisation and the humanisation of the ingroup. European Review of Social Psychology.

[224] Van Sittert, Lance and Sandra Swart. 2007. Canis Africanis: A Dog History of Southern Africa. In *Canis Africanis: A Dog History of Southern Africa*, ed. Lance van Sittert and Sandra Swart, 1–34. Brill.

[225] Van Sittert, Lance and Sandra Swart. 2003. Canis Africanis: A Dog History of Southern Africa. *South African Historical Journal* 48: 138–173.

[226] *Watson v SBV Services (Pty) Ltd*, ECEL4558-19.

[227] *Weitz v Southern Mapping Company (Pty) Ltd*, GAJB25903-19.

[228] Weston-Scheuber Kylie (2012). Gender and the Prohibition of Hate Speech. QUT Law & Justice Journal.

[229] Whillock, David. 1995. Symbols and Representation of Hate in Visual Discourse. In *Hate Speech*, ed. Rita Kirk Whillock and David Slayden, California: Sage Publications.

[230] White Carol Wayne (2017). Black Lives, Sacred Humanity, and the Racialization of Nature, or Why America Needs Religious Naturalism Today. American Journal of Theology & Philosophy.

[231] *WordSense Dictionary*. 2022. (a) 'hond’ From the Dutch ‘hond’ meaning ‘dog.’ Accessed 22 March 2022 from https://www.wordsense.eu/hond/. (b) 'ukuthakatha' Xhosa noun for witchcraft. Accessed 22 March 2022 from https://www.wordsense.eu/ukuthakatha/ and https://en.opentran.net/xhosa-english/ukuthakatha.html#translate.

[232] Woods, Vanessa and Brian Hare. 2019. Uncanny Valley of the Apes. In *Why We Love and Exploit Animals: Bridging Insights from Academia and Advocacy*, ed. Kristof Dhont and Gordon Hodson, 104–120. Routledge.

[233] Woodward Wendy (2008). The Animal Gaze: Animal Subjectivities in Southern African Narratives.

[234] *Wrobel v Southern Cape Business Systems Pty Ltd*, WEGE1802-18.

[235] Zevallos, Zuleyka. 2007. Sociology as ‘Other’: Representing Sociological Knowledge within a National Security Context. Defence Science and Technology Organisation, Edinburgh, Australia. Accessed on 15 April 2022. at https://researchbank.swinburne.edu.au/file/7fd04449-8be4-4973-bc9f-ee2c6b289fe5/1/PDF%20%28Published%20version%29.pdf.

[236] *Zulu English Dictionary*. Zulu noun for witchcraft from https://zulu.english-dictionary.help/zulu-to-english-meaning-ubuthakathi. Accessed 22 March 2022.

